# Supplementary material for: Cytokinetic abscission in Toxoplasma gondii is governed by protein phosphatase 2A and the daughter cell scaffold complex
Source: EMBO J. 2024 Jul 15;43(17):11. doi: 10.1038/s44318-024-00171-9 (PMC11377541; doi:10.1038/s44318-024-00171-9)
Supplement: Supplementary file 4 — Appendix [file 44318_2024_171_MOESM4_ESM.pdf]

APPENDIX

TABLE OF CONTENT

Appendix Figure S 1 2

Appendix Figure S 2 4

Appendix Figure S 3 5

Appendix Figure S 4 7

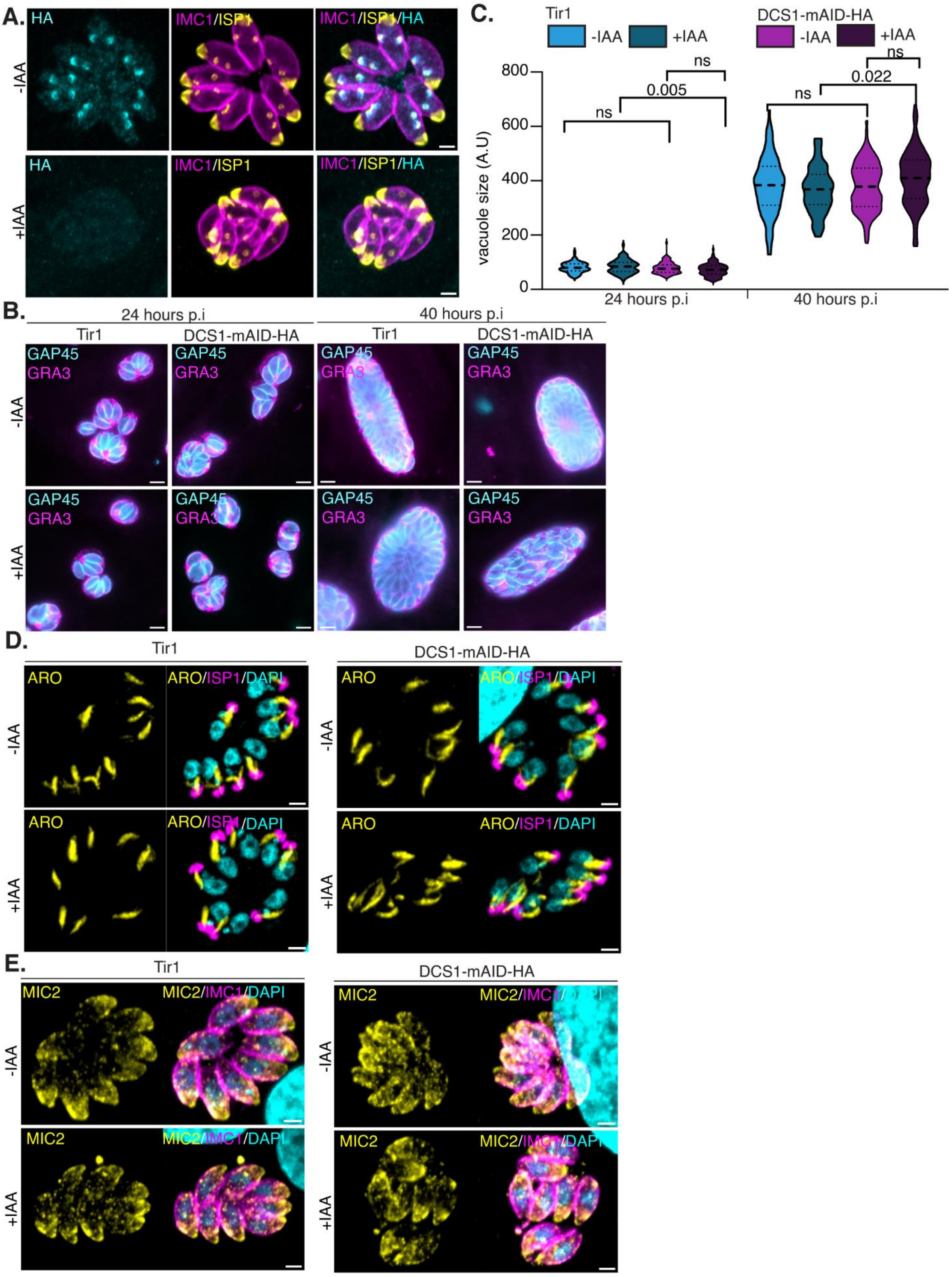

Appendix Figure S 1. DCS1 conditional depletion does not affect parasite growth and secretory organelle biogenesis.

A. IFA on intracellular DCS1-mAID-HA parasites showed that HA signal (cyan) became undetectable after 24h of IAA treatment. The apical cap and the IMC of the mother and daughter cells are detected with anti-ISP1 (yellow) and anti-IMC1 (magenta) antibodies. Scale bar = 2µm.

B. IFA of parasite culture at 24 and 40hpi (1st lytic cycle). Intact PV are visualized using anti-GRA3 antibodies and parasite are stained with anti-GAP45 antibodies. Scale bar = 5µm. Image representative of three independent biological replicates.

C. Quantification of the vacuole size determined using GRA3 staining at 24 and 40hpi. Two-way ANOVA followed by Tukey's multiple comparison was used to test differences between groups (mean ± SD; n=3 biologically independent experiments).

D. IFA of intracellular parasites stained with ARO and ISP1 antibodies to visualize the rhoptry secretory organelle and the IMC apical cap of individual parasite. Scale bar = 2µm.

E. IFA of intracellular parasites stained with MIC2 and IMC1 antibodies to visualize the microneme secretory organelle and the IMC of individual parasite. Scale bar = 2µm.

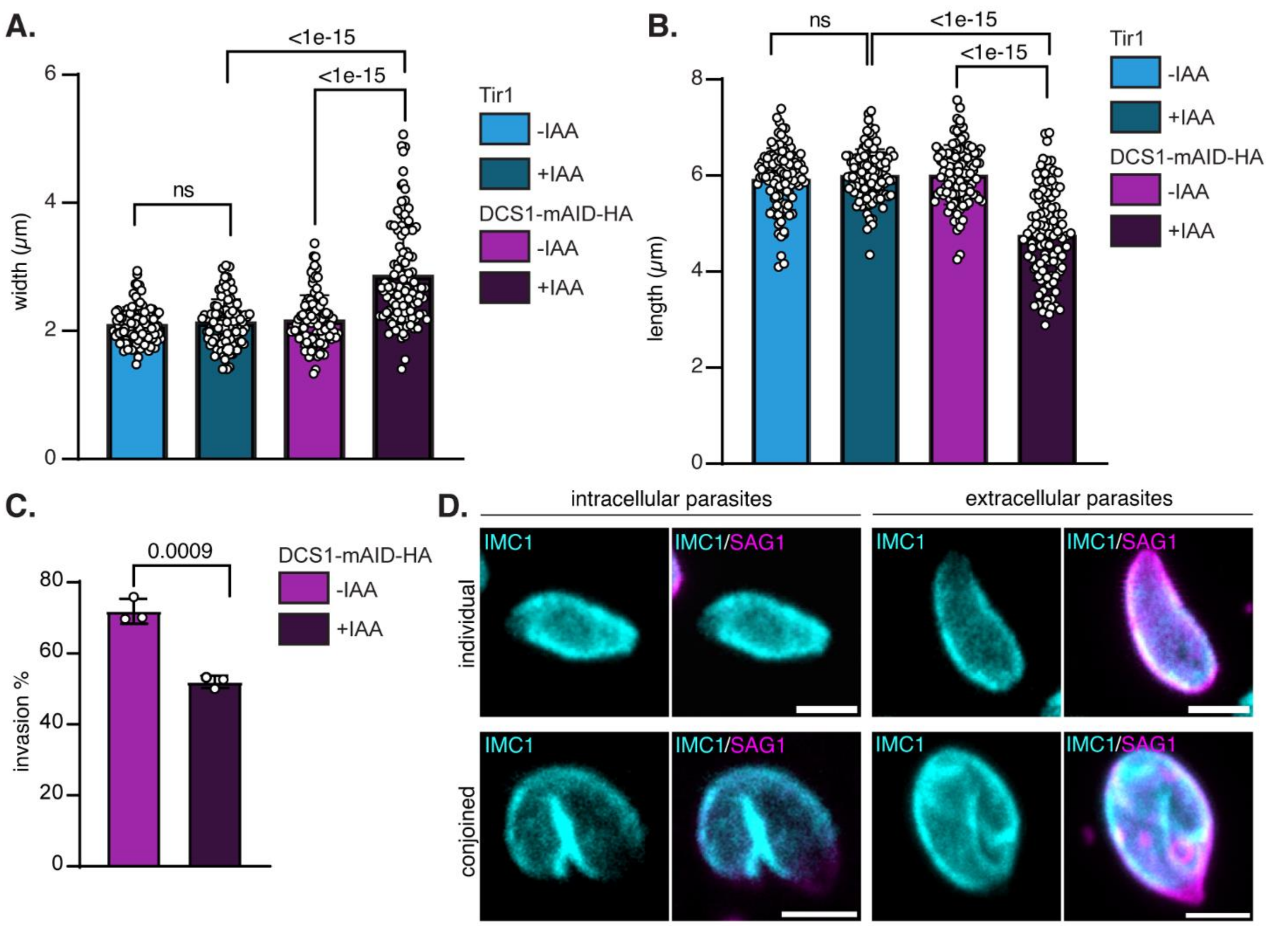

Appendix Figure S 2. Aberrant morphology of DCS1-depleted parasites.

A. Graph showing the width of each individual parasites measured using SAG1 staining of one biological replicate. One-way ANOVA followed by Tukey's multiple comparison was used to test differences between groups (mean ± SD). Experiment was performed in three independent biological replicates showing the same tendency.

B. Graph showing the length of each individual parasites measured using SAG1 staining of one biological replicate. One-way ANOVA followed by Tukey's multiple comparison was used to test differences between groups (mean ± SD). Experiment was performed in three independent biological replicates showing the same tendency.

C. Invasion assay graph showing the percentage of intracellular parasite 15 min post-infection. One-way ANOVA followed by Tukey's multiple comparison was used to test differences between groups (mean ± SD; n=3 biologically independent experiments).

D. IFA of the 15 min invasion assay performed on DCS1-mAID-HA parasites treated or not with IAA for 18h. The images depicted are representative of the intracellular and extracellular parasites observed in this experiment. The SAG1 staining was used to stain that the parasite are intracellular and IMC1 staining was used to identify the parasite morphology. Scale bar = 2µm.

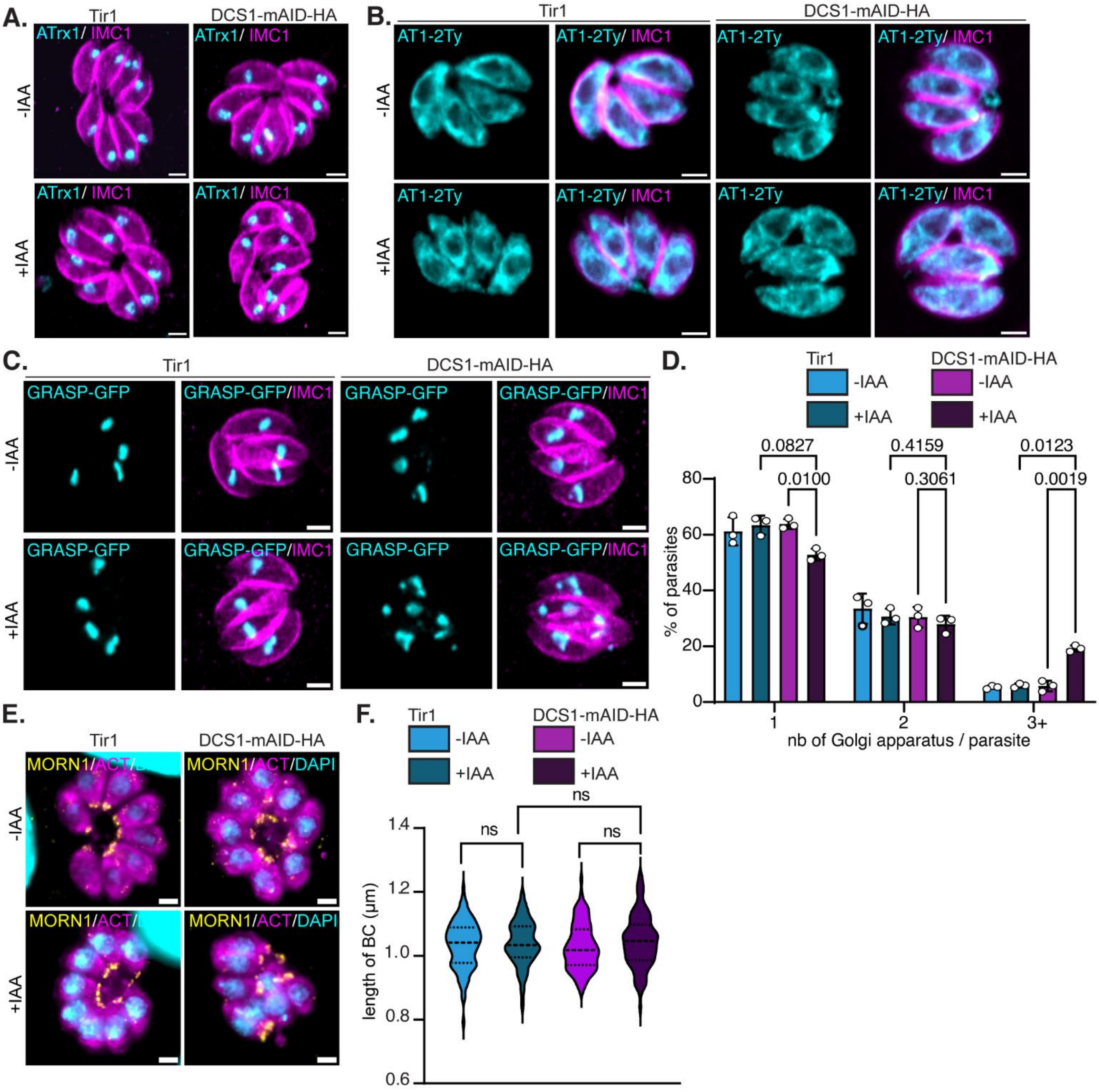

Appendix Figure S 3. DCS1 depletion leads to a moderate Golgi apparatus fragmentation.

A. IFA of intracellular parasites showing no defect in apicoplast segregation between DCs under DCS1 depletion. ATrx1 (cyan) and IMC1 (magenta) antibodies were used to visualize the apicoplast and the IMC of each parasite. Scale bar = 2µm.

B. IFA of intracellular parasites showing no defect in the ER ultrastructure under DCS1 depletion. AT1-2Ty transiently expressed (cyan) and IMC1 antibodies (magenta) were used to visualize the ER and the IMC of each parasite. Scale bar = 2µm.

C. IFA of intracellular parasites showing a moderated Golgi fragmentation under DCS1 depletion. GRASP-GFP transiently expressed (cyan) and IMC1 antibodies (magenta) were used to visualize the Golgi apparatus and the IMC of each parasite. Scale bar = 2µm.

D. Quantification of the number of GRASP-GFP puncta in each individual parasite. Two-way ANOVA followed by Tukey's multiple comparison was used to test differences between groups (mean ± SD; n= 3 independent biological replicates).

E. IFA of intracellular parasites stained with MORN1 (yellow) and Actin (magenta) antibodies. MORN1 is used to visualize the BC of each individual parasites. Scale bar = 2µm.

F. Quantification of the length of the BC (µm) of each individual parasite using the MORN1 antibody staining. Two-way ANOVA followed by Tukey's multiple comparison was used to test differences between groups (mean ± SD; n= 4 independent biological replicates).

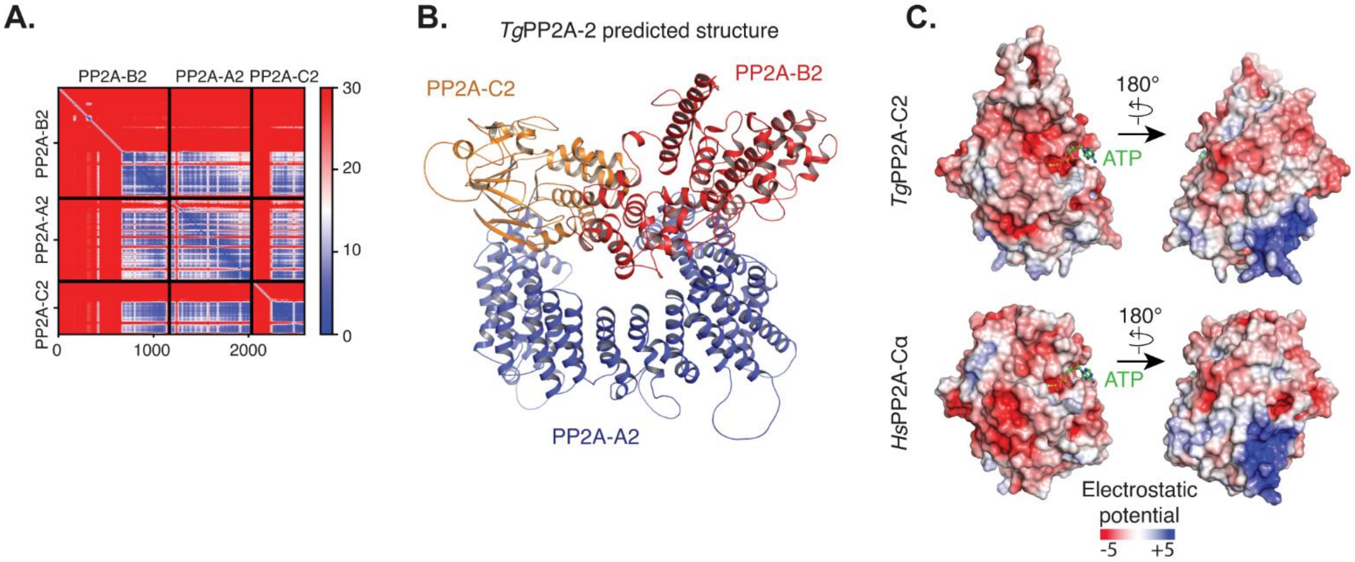

Appendix Figure S 4. Structural analysis of the predicted TgPP2A-2 holoenzyme.

A. Predicted Aligned Error (PAE) plot of the AlphaFold prediction for the trimeric complex.

B. Model of *T.gondii* PP2A-2 holoenzyme with each of the three subunits coloured differently.

C. Surface representation of TgPP2A-C2 (top) and human PP2A-Calpha (bottom) with modelled ATPγS positioned based on human complex (pdb: 4lac). Surface is coloured according to its electrostatic potential.
