# Supplementary figures and images for: Cytokinetic abscission in Toxoplasma gondii is governed by protein phosphatase 2A and the daughter cell scaffold complex
Source: EMBO J. 2024 Jul 15;43(17):11. doi: 10.1038/s44318-024-00171-9 (PMC11377541; doi:10.1038/s44318-024-00171-9)

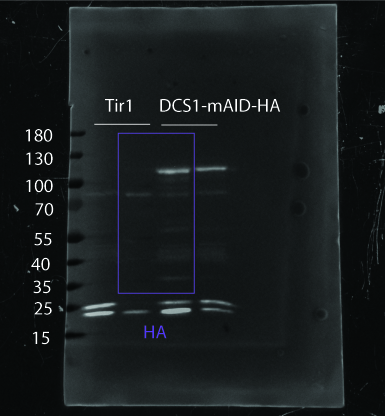

Supplement: Supplementary file 10 — Source data Fig. 1 [file 44318_2024_171_MOESM10_ESM.zip › Figure 1/1A/WB_DSC1mAIDHA_HA.tif]

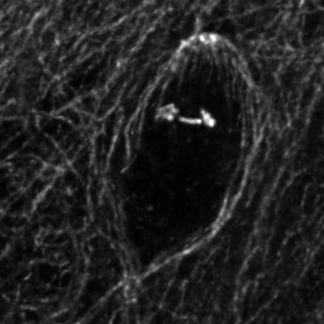

Supplement: Supplementary file 10 — Source data Fig. 1 [file 44318_2024_171_MOESM10_ESM.zip › Figure 1/1C/1-AcTub-MAX.tif]

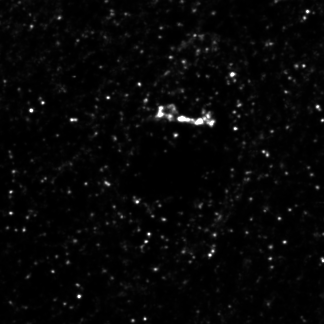

Supplement: Supplementary file 10 — Source data Fig. 1 [file 44318_2024_171_MOESM10_ESM.zip › Figure 1/1C/1-HA-MAX.tif]

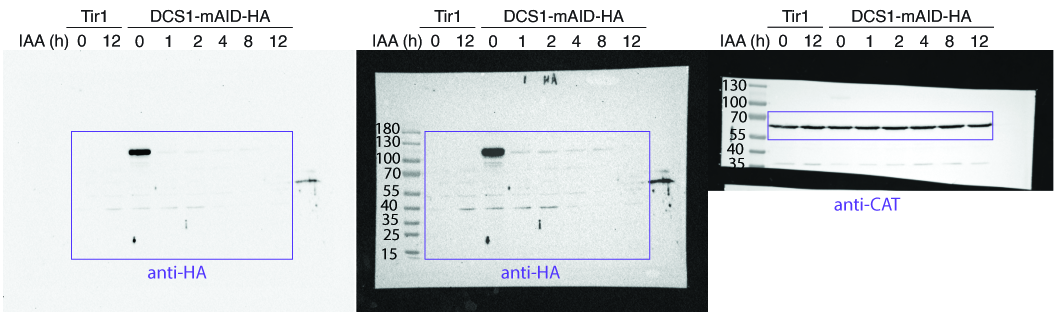

Supplement: Supplementary file 11 — Source data Fig. 2 [file 44318_2024_171_MOESM11_ESM.zip › Figure 2/2A/WB_DSC1mAIDHA_HA_CAT_KD.tif]

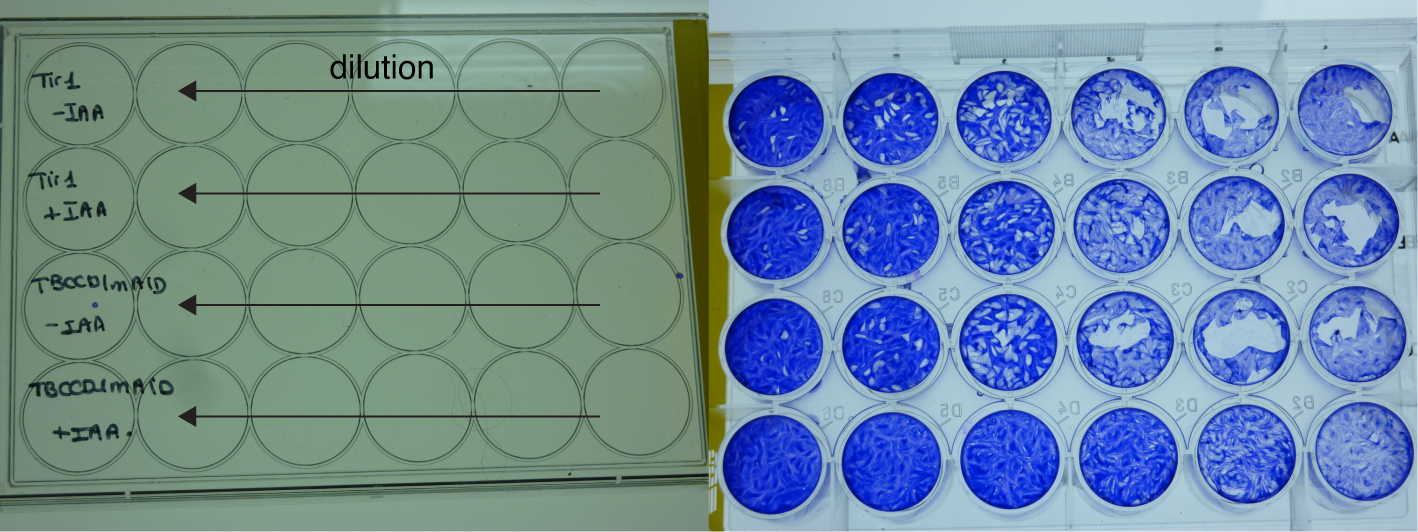

Supplement: Supplementary file 11 — Source data Fig. 2 [file 44318_2024_171_MOESM11_ESM.zip › Figure 2/2B/DCS1_PlaqueAssay.tif]

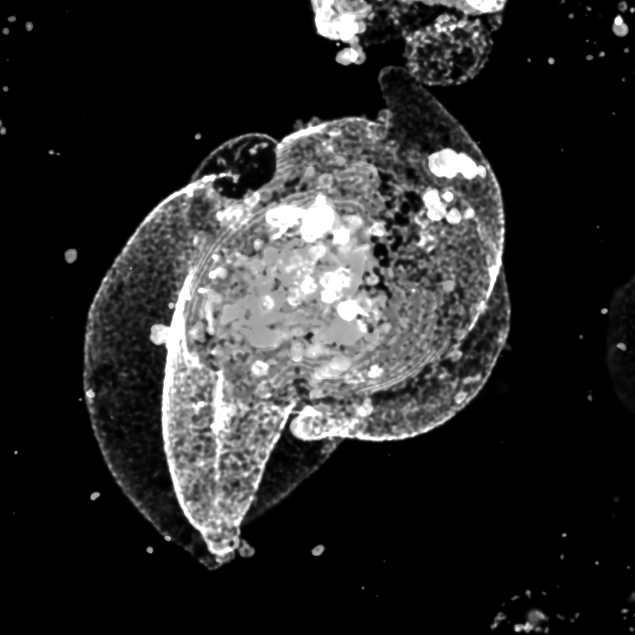

Supplement: Supplementary file 12 — Source data Fig. 3 [file 44318_2024_171_MOESM12_ESM.zip › Figure 3/3C/SAG1_MAX_DCS1+IAA_extracellular.tif]

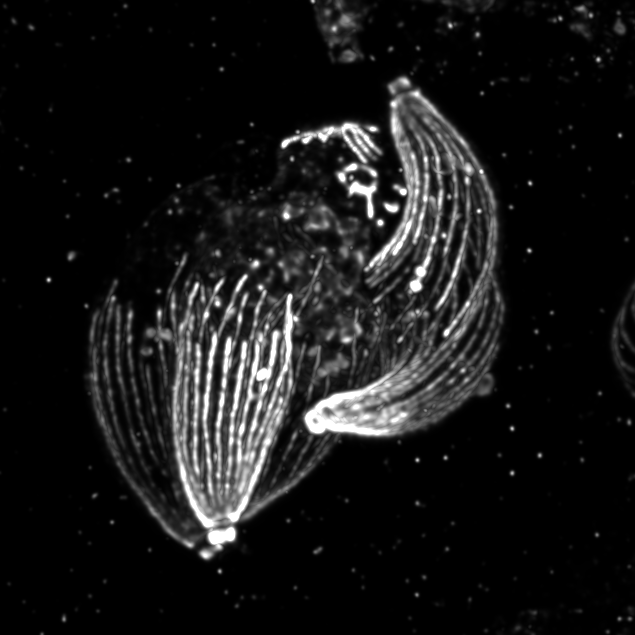

Supplement: Supplementary file 12 — Source data Fig. 3 [file 44318_2024_171_MOESM12_ESM.zip › Figure 3/3C/Tub_MAX_DCS1+IAA_extracellular.tif]

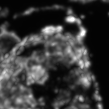

Supplement: Supplementary file 12 — Source data Fig. 3 [file 44318_2024_171_MOESM12_ESM.zip › Figure 3/3D/SAG1-MAX_protruded.tif]

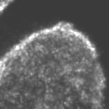

Supplement: Supplementary file 12 — Source data Fig. 3 [file 44318_2024_171_MOESM12_ESM.zip › Figure 3/3D/SAG1-MAX_retracted.tif]

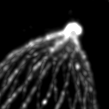

Supplement: Supplementary file 12 — Source data Fig. 3 [file 44318_2024_171_MOESM12_ESM.zip › Figure 3/3D/Tub-MAX_protruded.tif]

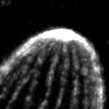

Supplement: Supplementary file 12 — Source data Fig. 3 [file 44318_2024_171_MOESM12_ESM.zip › Figure 3/3D/Tub-MAX_retracted.tif]

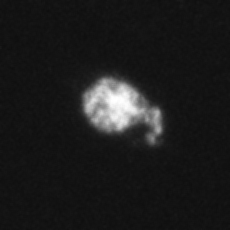

Supplement: Supplementary file 12 — Source data Fig. 3 [file 44318_2024_171_MOESM12_ESM.zip › Figure 3/3F/i) individual_nondividing/DAPI-MAX_Image 6.tif]

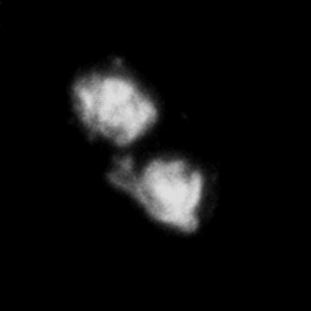

Supplement: Supplementary file 12 — Source data Fig. 3 [file 44318_2024_171_MOESM12_ESM.zip › Figure 3/3F/ii) individual_dividing/DAPI-MAX_Image 9.tif]

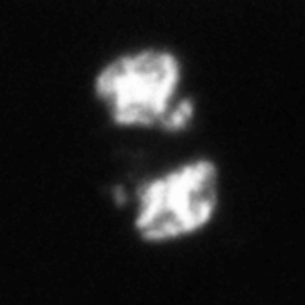

Supplement: Supplementary file 12 — Source data Fig. 3 [file 44318_2024_171_MOESM12_ESM.zip › Figure 3/3F/iii) conjoined_upperpanel/DAPI-conjoined.tif]

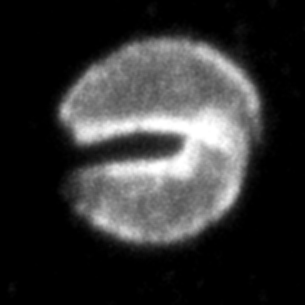

Supplement: Supplementary file 12 — Source data Fig. 3 [file 44318_2024_171_MOESM12_ESM.zip › Figure 3/3F/iii) conjoined_upperpanel/IMC1-conjoined.tif]

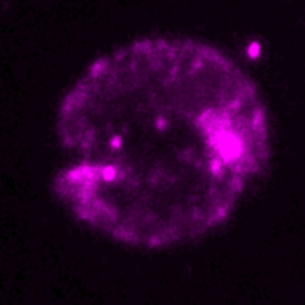

Supplement: Supplementary file 12 — Source data Fig. 3 [file 44318_2024_171_MOESM12_ESM.zip › Figure 3/3F/iii) conjoined_upperpanel/SAG1-conjoined.tif]

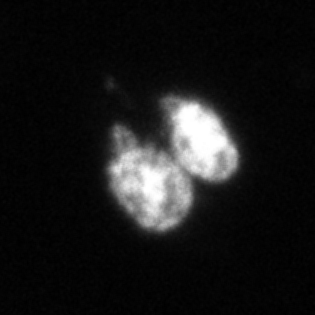

Supplement: Supplementary file 12 — Source data Fig. 3 [file 44318_2024_171_MOESM12_ESM.zip › Figure 3/3F/iv) conjoined_middlepanel/DAPI-conjoined.tif]

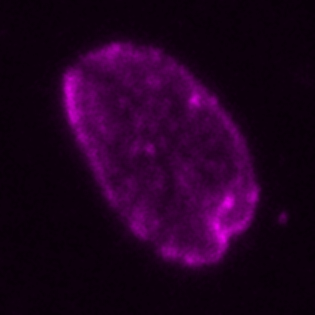

Supplement: Supplementary file 12 — Source data Fig. 3 [file 44318_2024_171_MOESM12_ESM.zip › Figure 3/3F/iv) conjoined_middlepanel/SAG1-conjoined.tif]

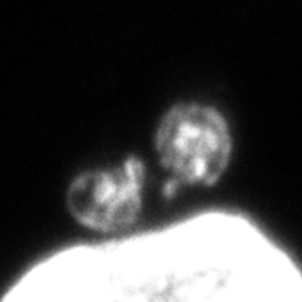

Supplement: Supplementary file 12 — Source data Fig. 3 [file 44318_2024_171_MOESM12_ESM.zip › Figure 3/3F/v) coma_lowerpanel/DAPI-coma-shaped.tif]

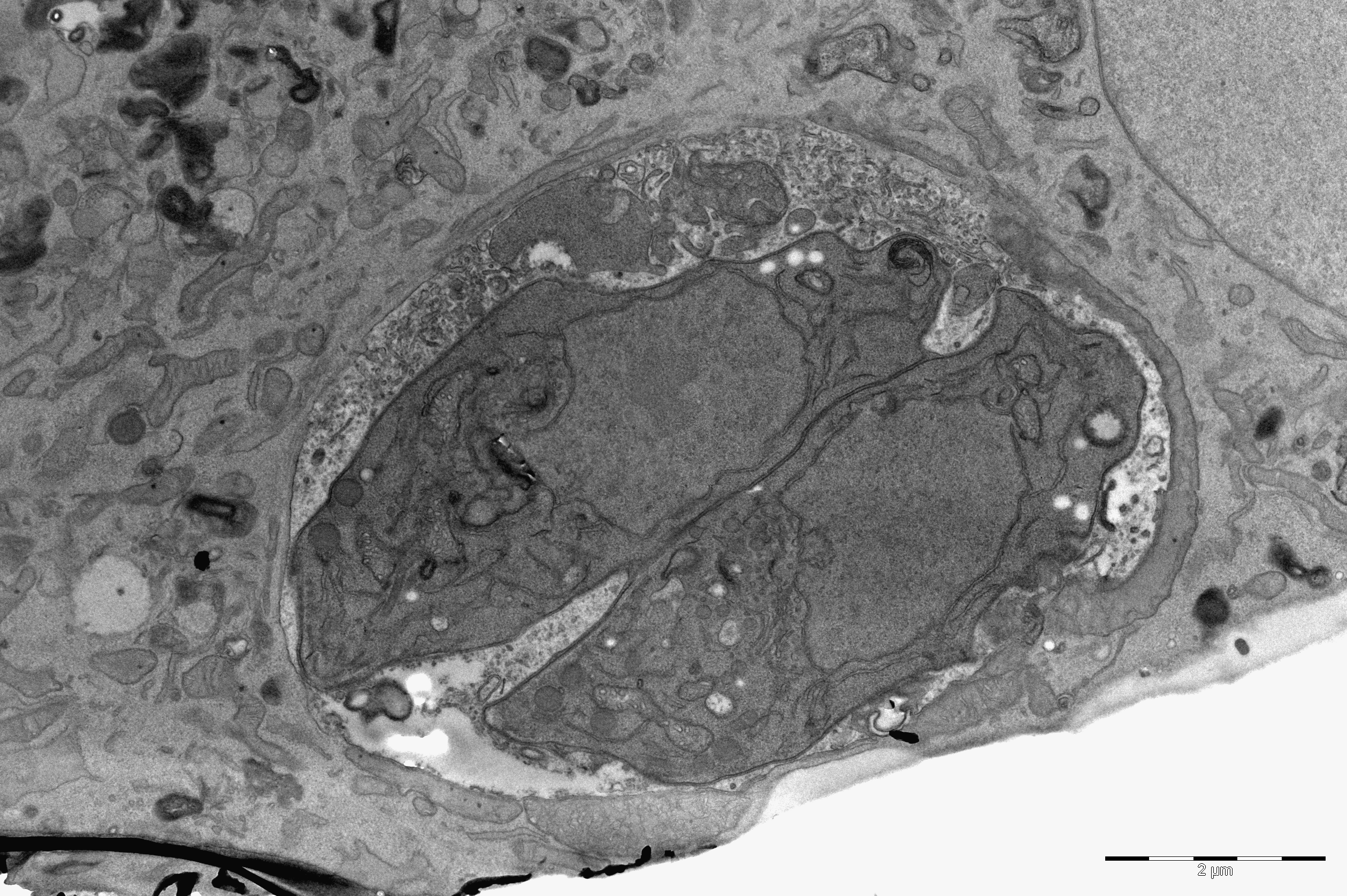

Supplement: Supplementary file 13 — Source data Fig. 4 [file 44318_2024_171_MOESM13_ESM.zip › Figure 4/4D/EM_DCS1+IAA.tif]

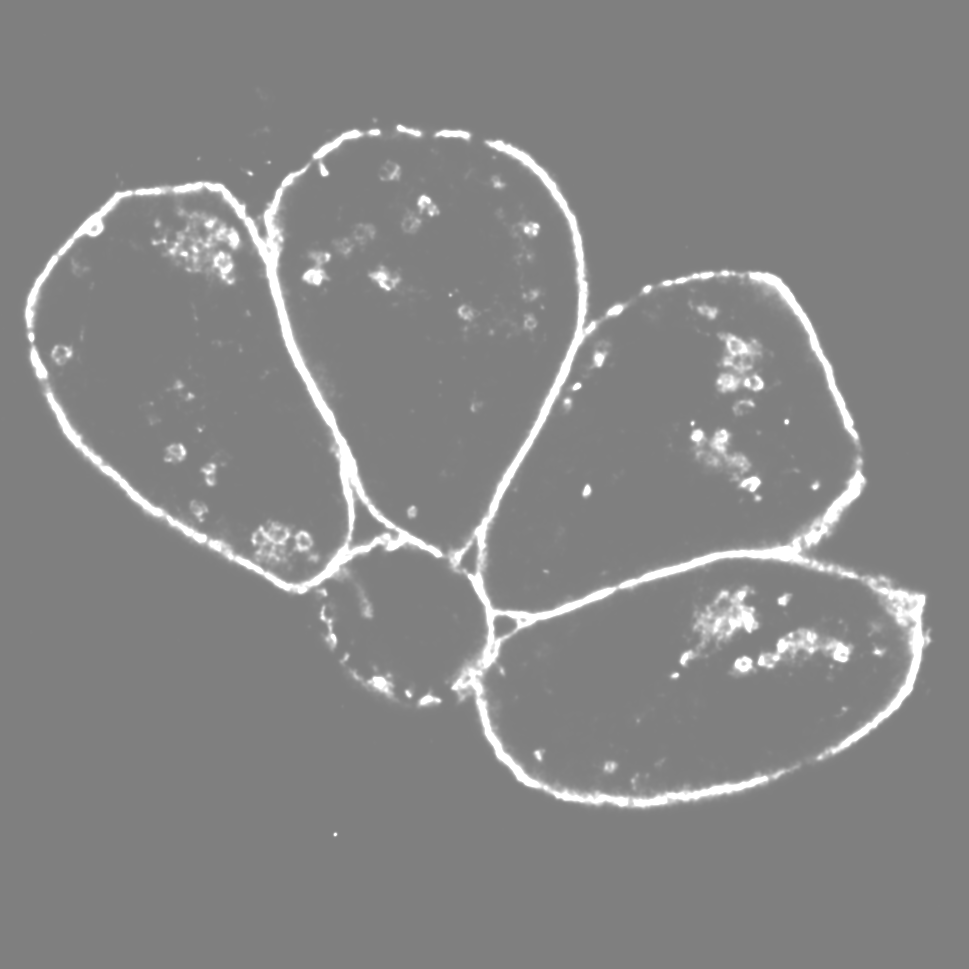

Supplement: Supplementary file 13 — Source data Fig. 4 [file 44318_2024_171_MOESM13_ESM.zip › Figure 4/4E/i)Tir1/SAG1_Tir1.tif]

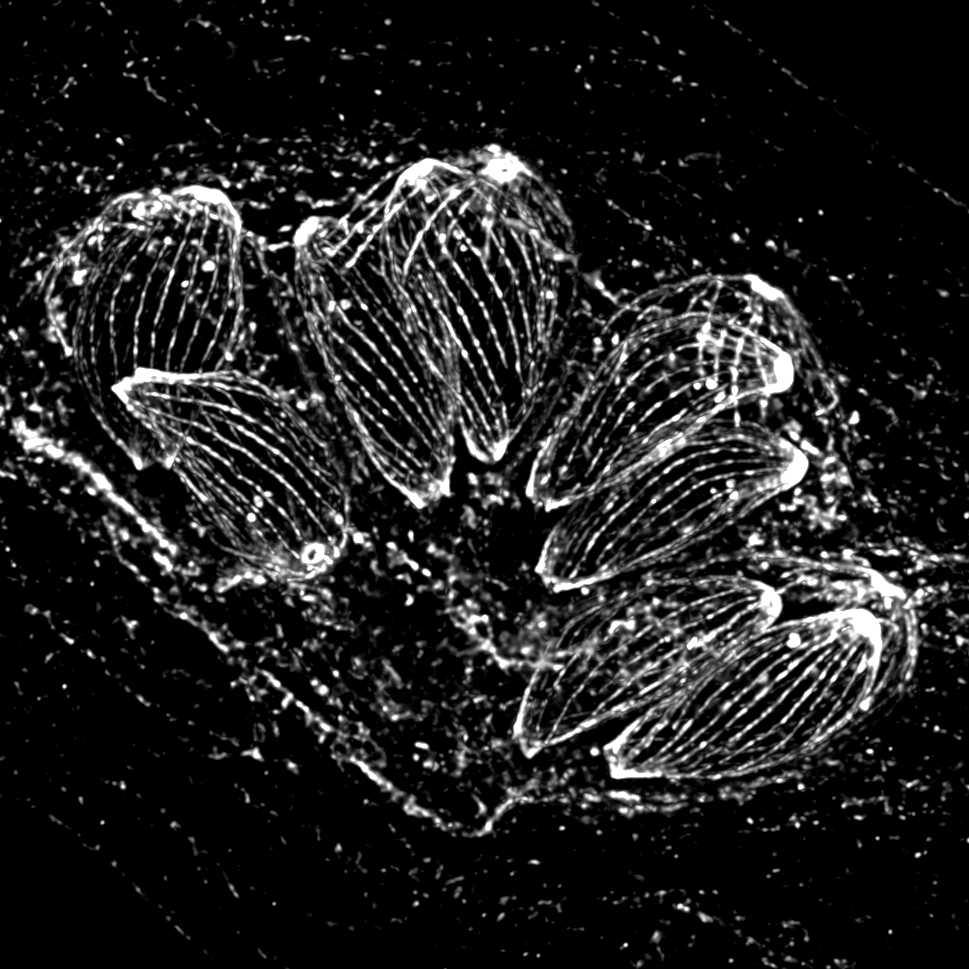

Supplement: Supplementary file 13 — Source data Fig. 4 [file 44318_2024_171_MOESM13_ESM.zip › Figure 4/4E/i)Tir1/Tub_Tir1.tif]

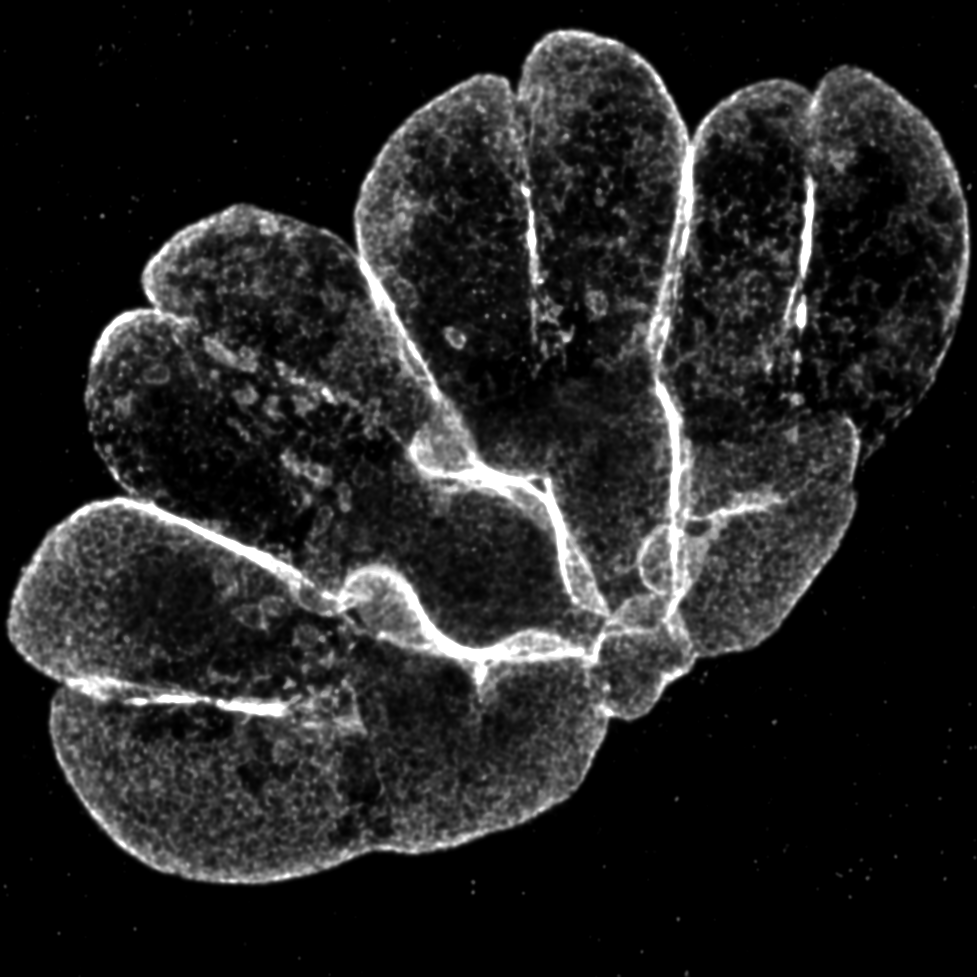

Supplement: Supplementary file 13 — Source data Fig. 4 [file 44318_2024_171_MOESM13_ESM.zip › Figure 4/4E/ii)Tir1/SAG1_Tir1.tif]

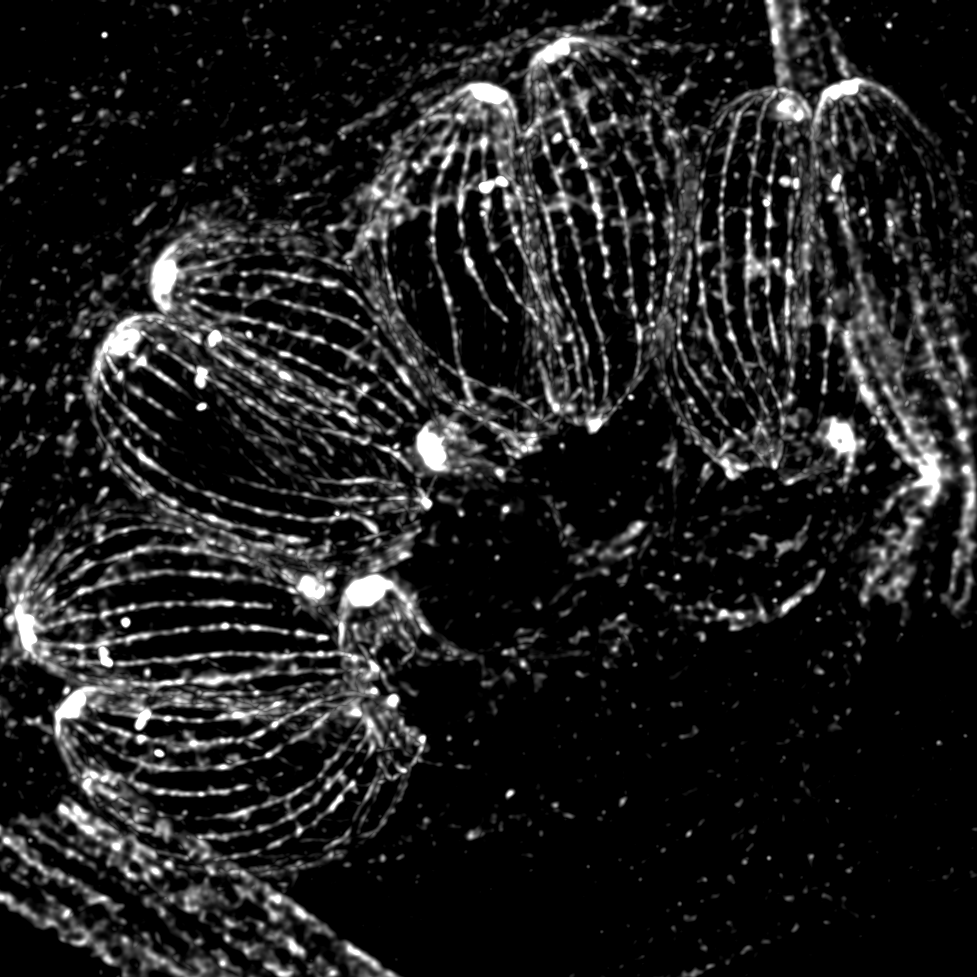

Supplement: Supplementary file 13 — Source data Fig. 4 [file 44318_2024_171_MOESM13_ESM.zip › Figure 4/4E/ii)Tir1/Tub_Tir1.tif]

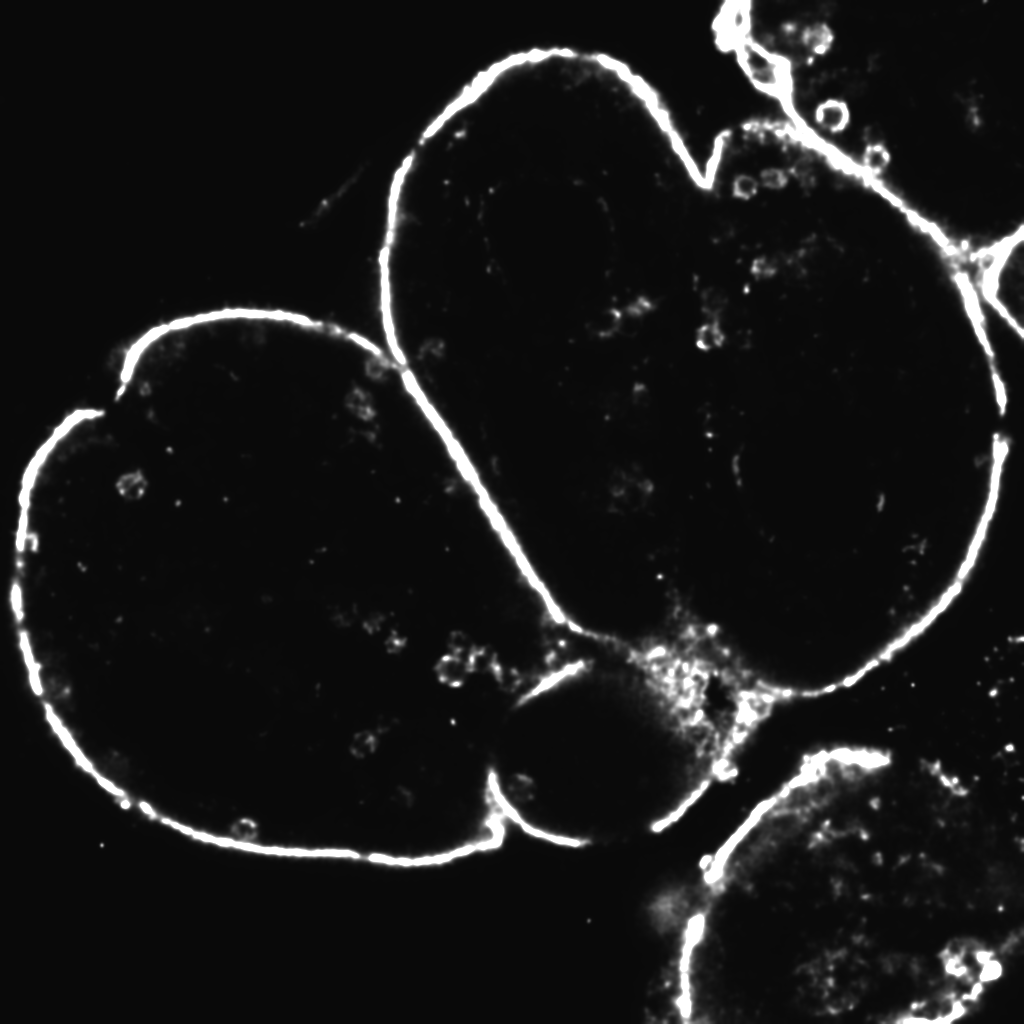

Supplement: Supplementary file 13 — Source data Fig. 4 [file 44318_2024_171_MOESM13_ESM.zip › Figure 4/4E/iii)DCS1-mAID-HA/SAG1_DCS1.tif]

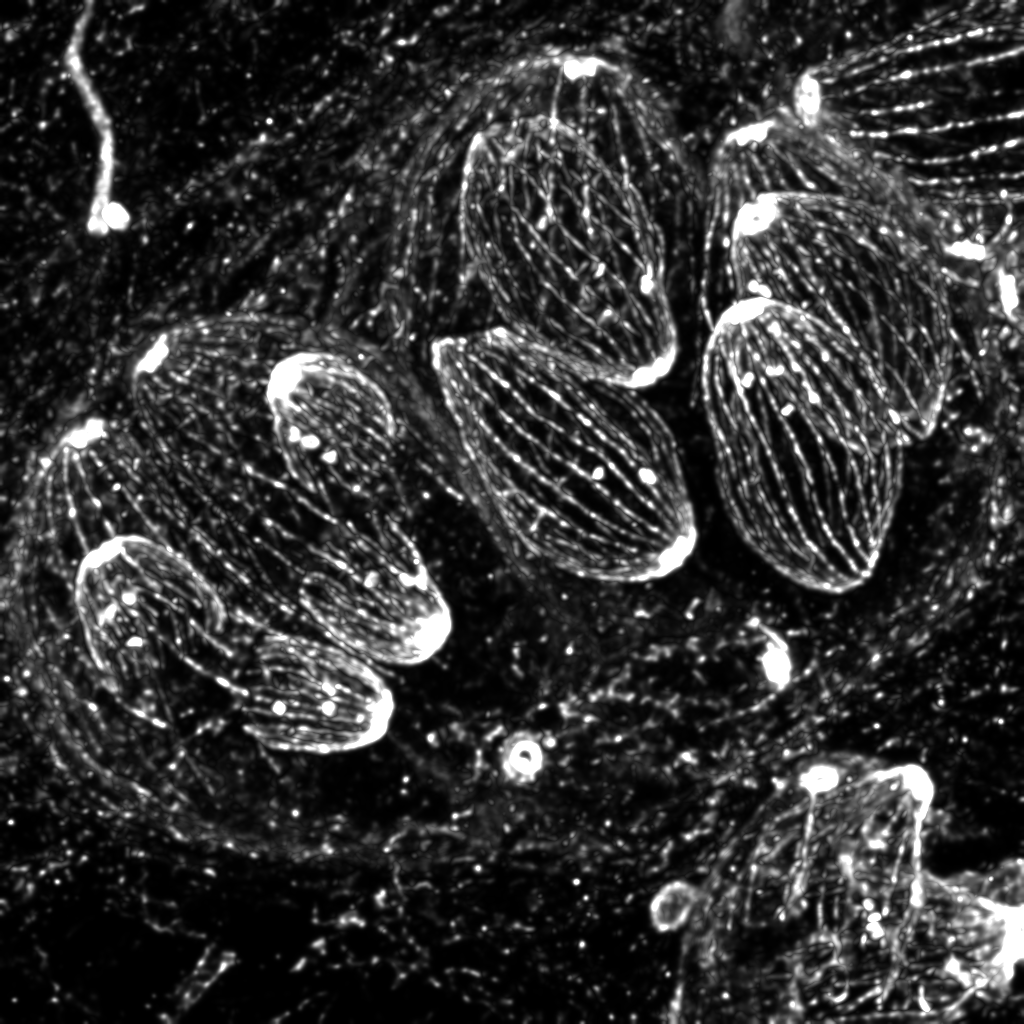

Supplement: Supplementary file 13 — Source data Fig. 4 [file 44318_2024_171_MOESM13_ESM.zip › Figure 4/4E/iii)DCS1-mAID-HA/Tub_DCS1.tif]

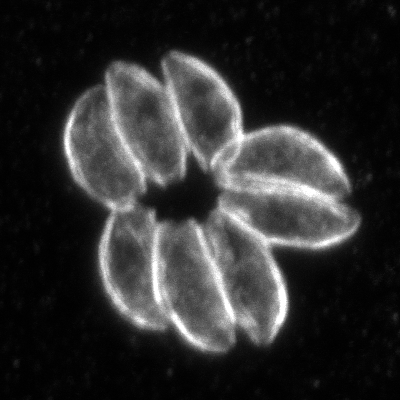

Supplement: Supplementary file 14 — Source data Fig. 5 [file 44318_2024_171_MOESM14_ESM.zip › Figure 5/5C/GAP45-Tir1+IAA.tif]

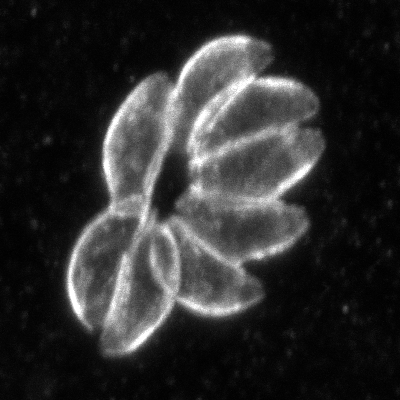

Supplement: Supplementary file 14 — Source data Fig. 5 [file 44318_2024_171_MOESM14_ESM.zip › Figure 5/5C/GAP45-Tir1-IAA.tif]

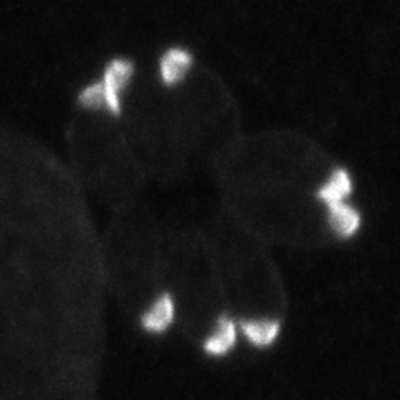

Supplement: Supplementary file 14 — Source data Fig. 5 [file 44318_2024_171_MOESM14_ESM.zip › Figure 5/5C/ISP1-Tir1+IAA.tif]

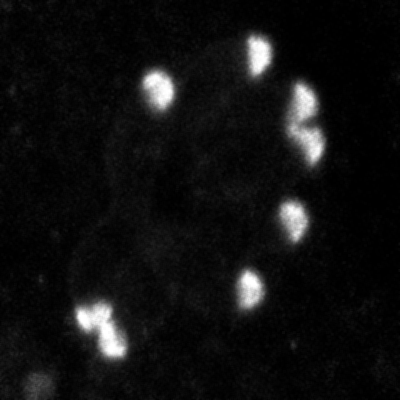

Supplement: Supplementary file 14 — Source data Fig. 5 [file 44318_2024_171_MOESM14_ESM.zip › Figure 5/5C/ISP1-Tir1-IAA.tif]

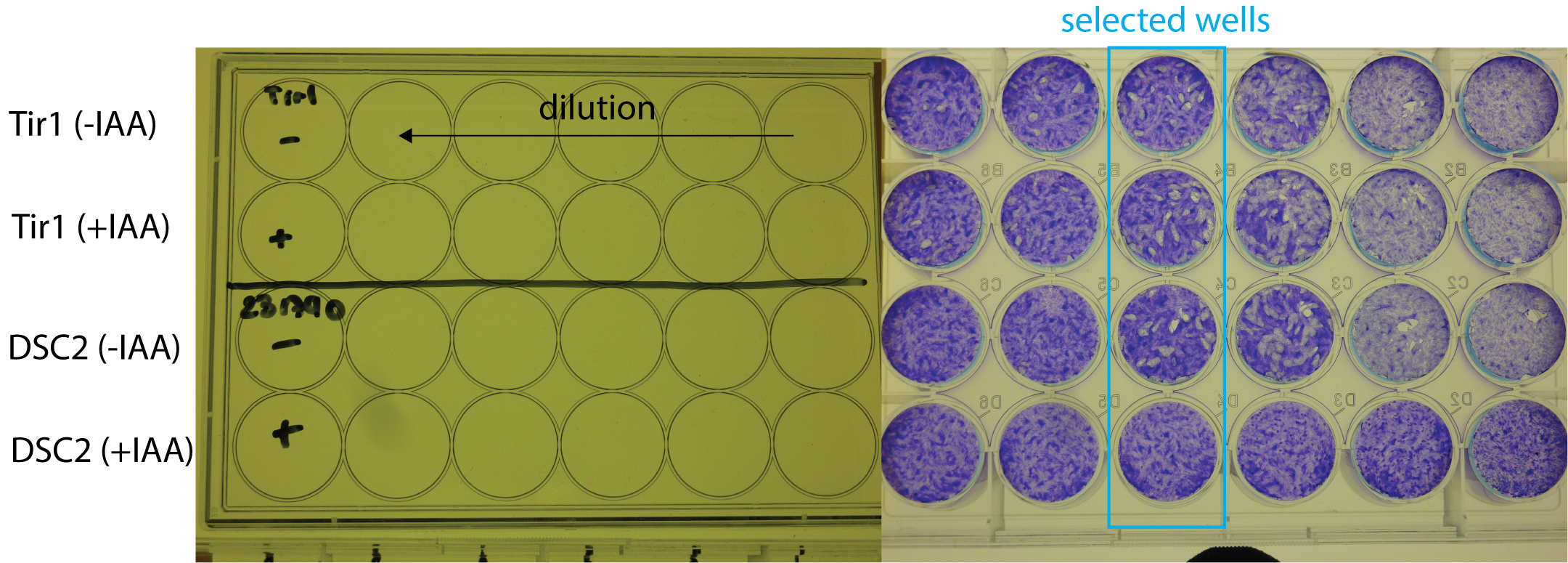

Supplement: Supplementary file 15 — Source data Fig. 6 [file 44318_2024_171_MOESM15_ESM.zip › Figure 6/6D/DCS2_PlaqueAssay.tif]

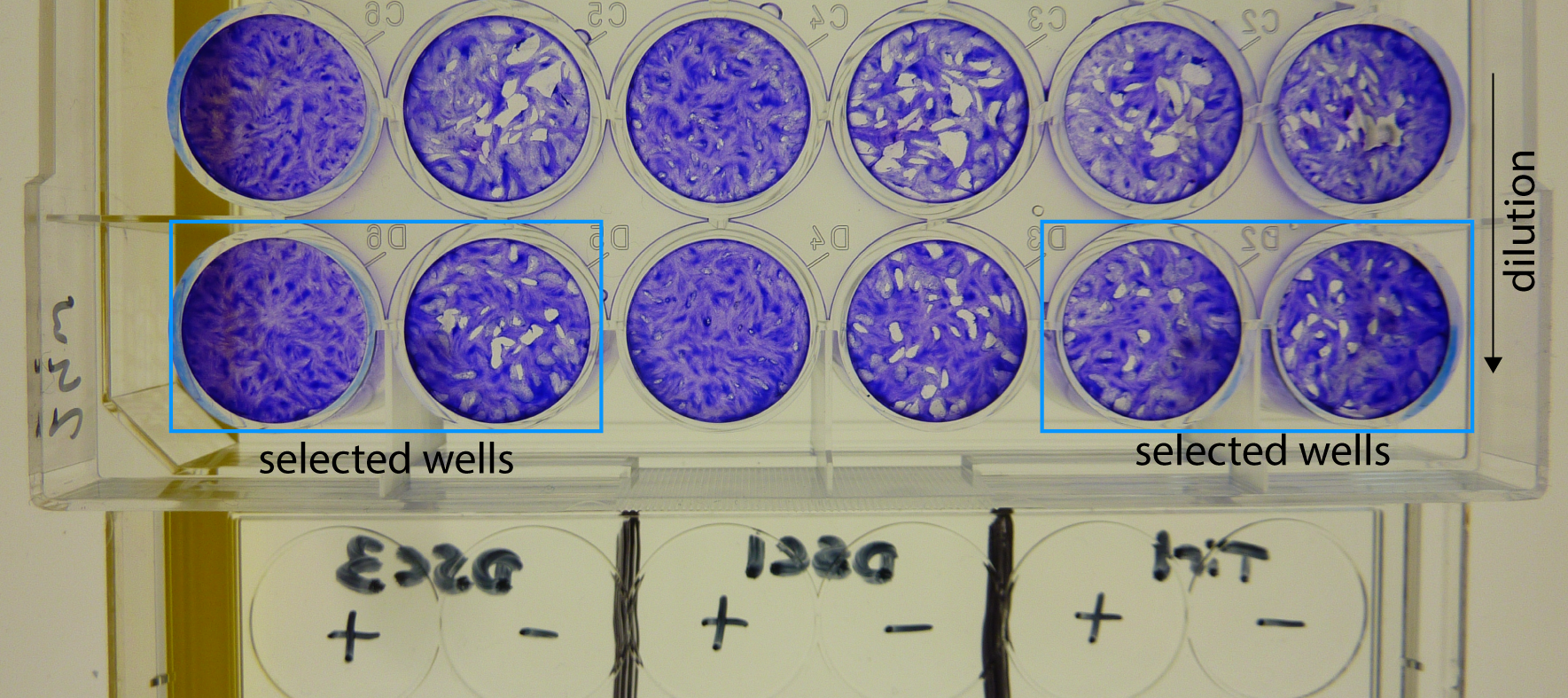

Supplement: Supplementary file 15 — Source data Fig. 6 [file 44318_2024_171_MOESM15_ESM.zip › Figure 6/6D/PP2AB2_PlaqueAssay-01.tif]

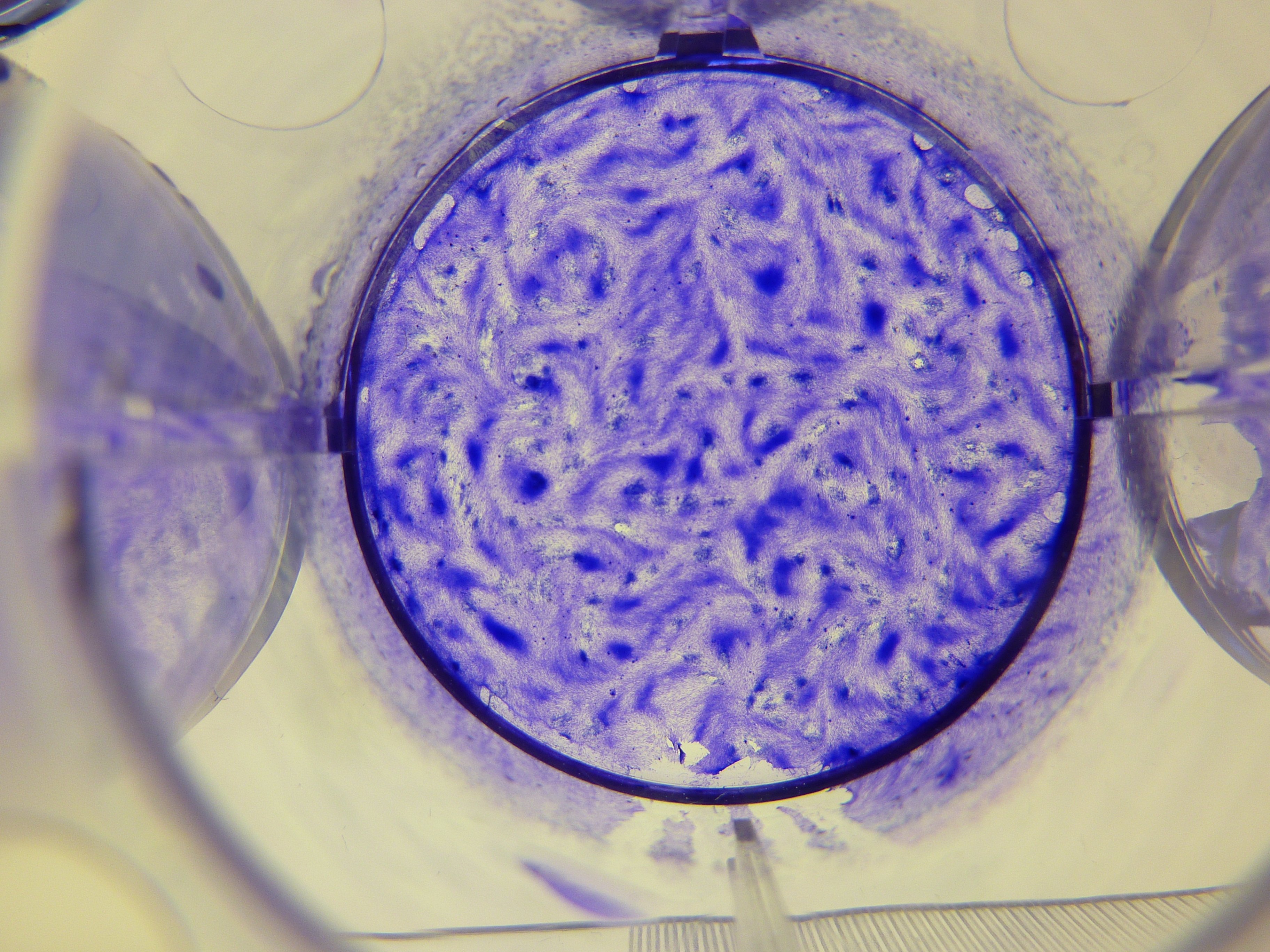

Supplement: Supplementary file 16 — Source data Fig. 7 [file 44318_2024_171_MOESM16_ESM.zip › Figure 7/7F/DCS1+IAA.JPG]

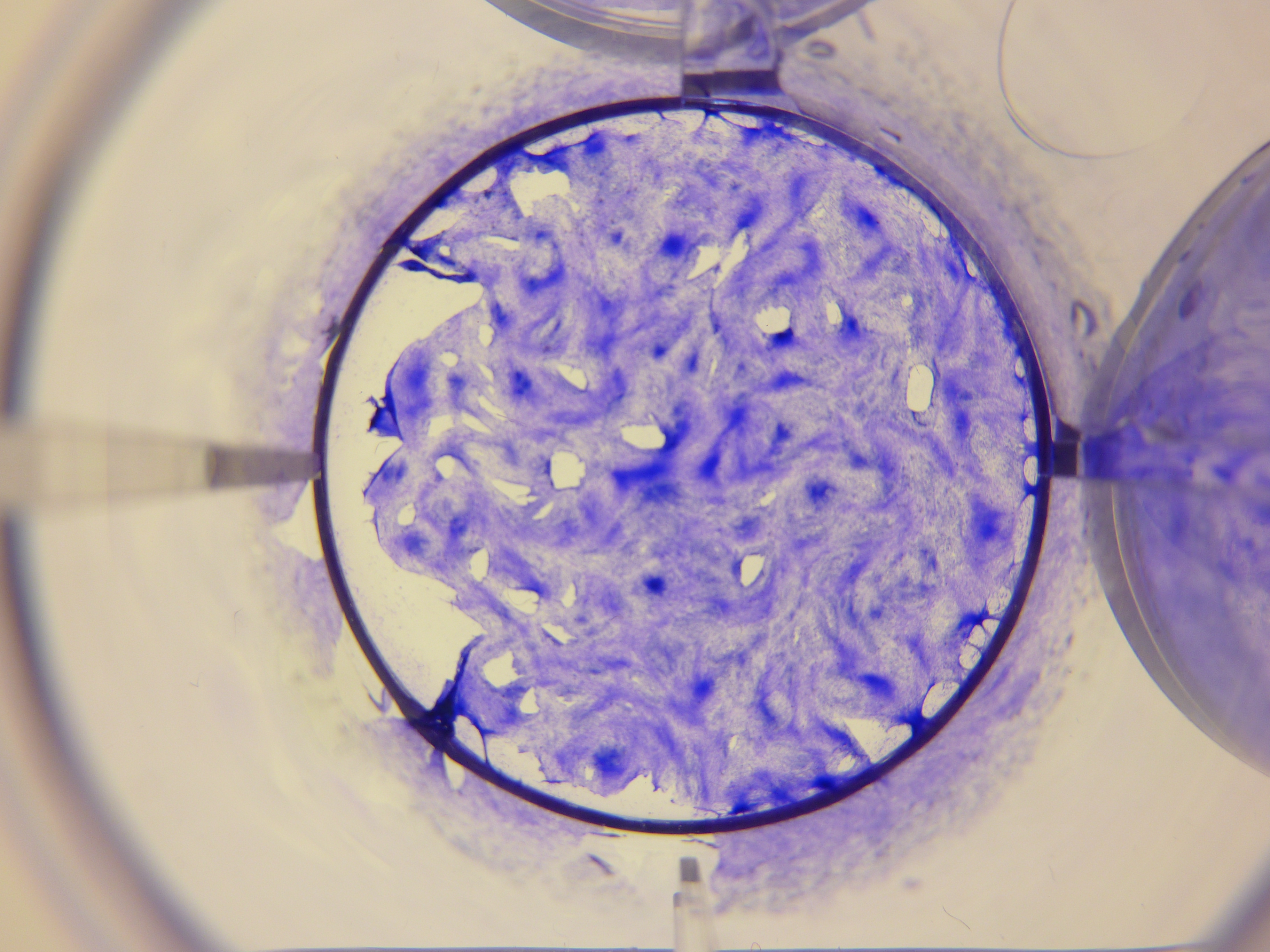

Supplement: Supplementary file 16 — Source data Fig. 7 [file 44318_2024_171_MOESM16_ESM.zip › Figure 7/7F/DCS1-IAA.JPG]

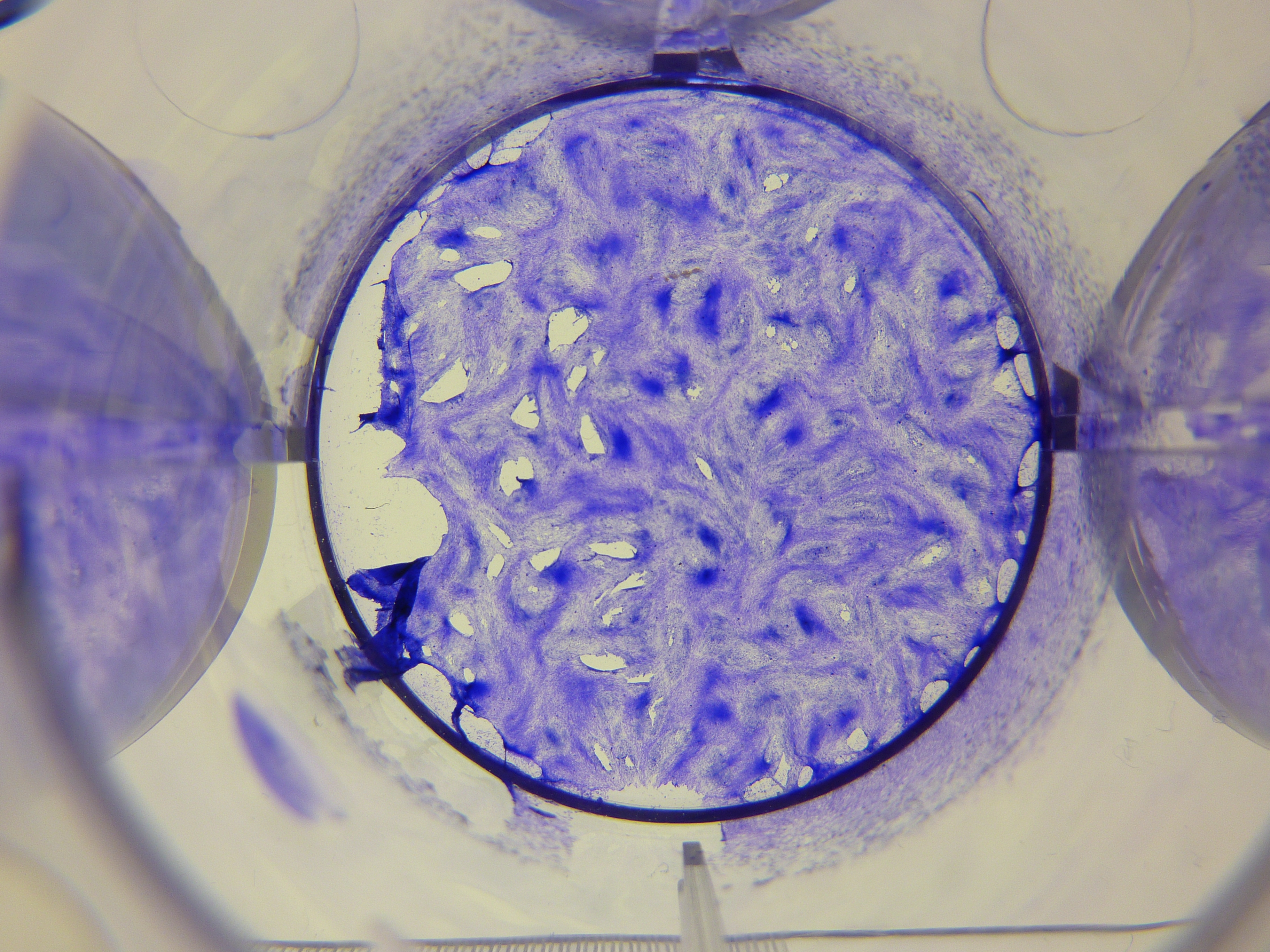

Supplement: Supplementary file 16 — Source data Fig. 7 [file 44318_2024_171_MOESM16_ESM.zip › Figure 7/7F/DCS1_24IAA_wo.JPG]

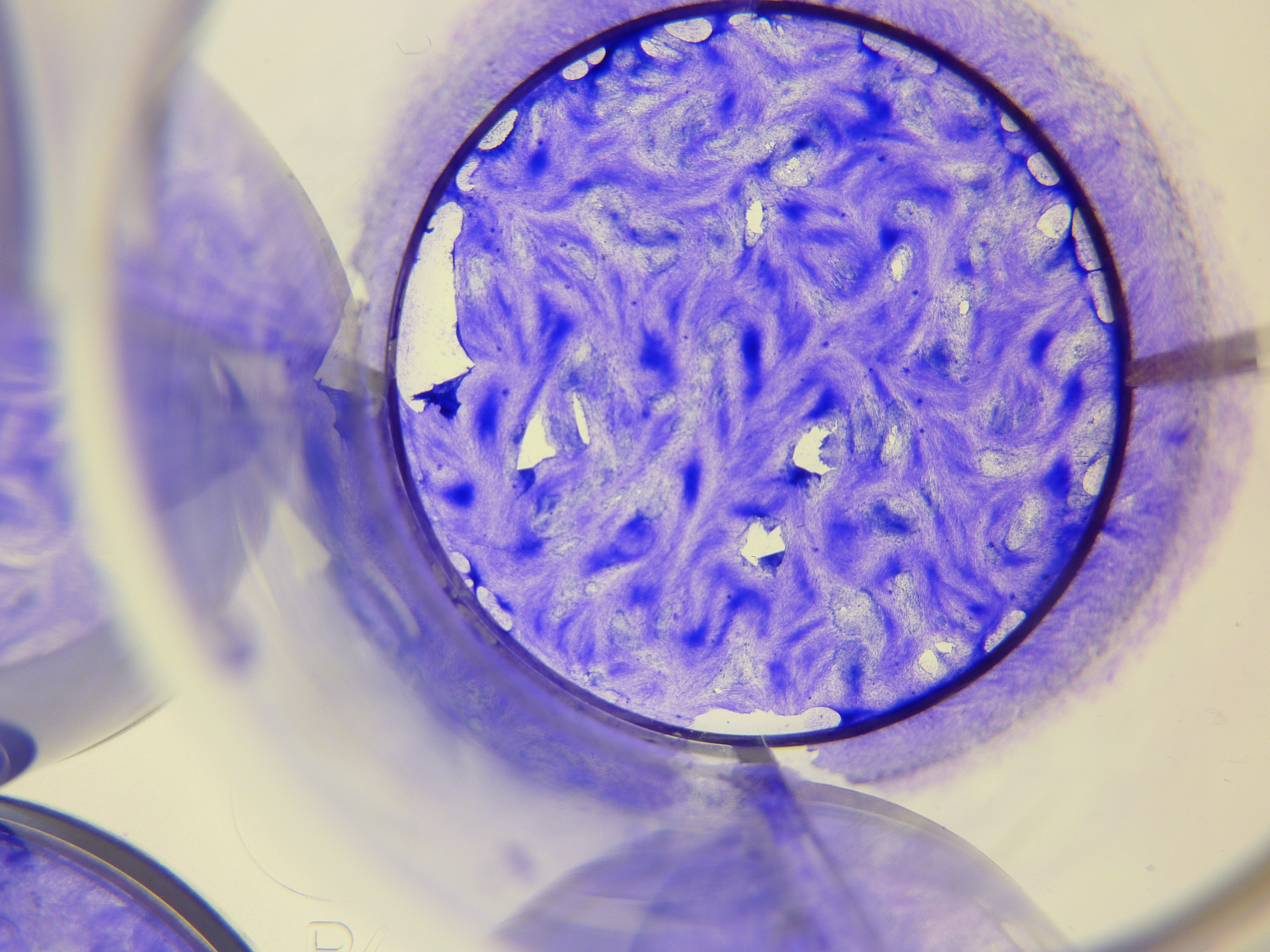

Supplement: Supplementary file 16 — Source data Fig. 7 [file 44318_2024_171_MOESM16_ESM.zip › Figure 7/7F/DCS1_48IAA_wo.JPG]

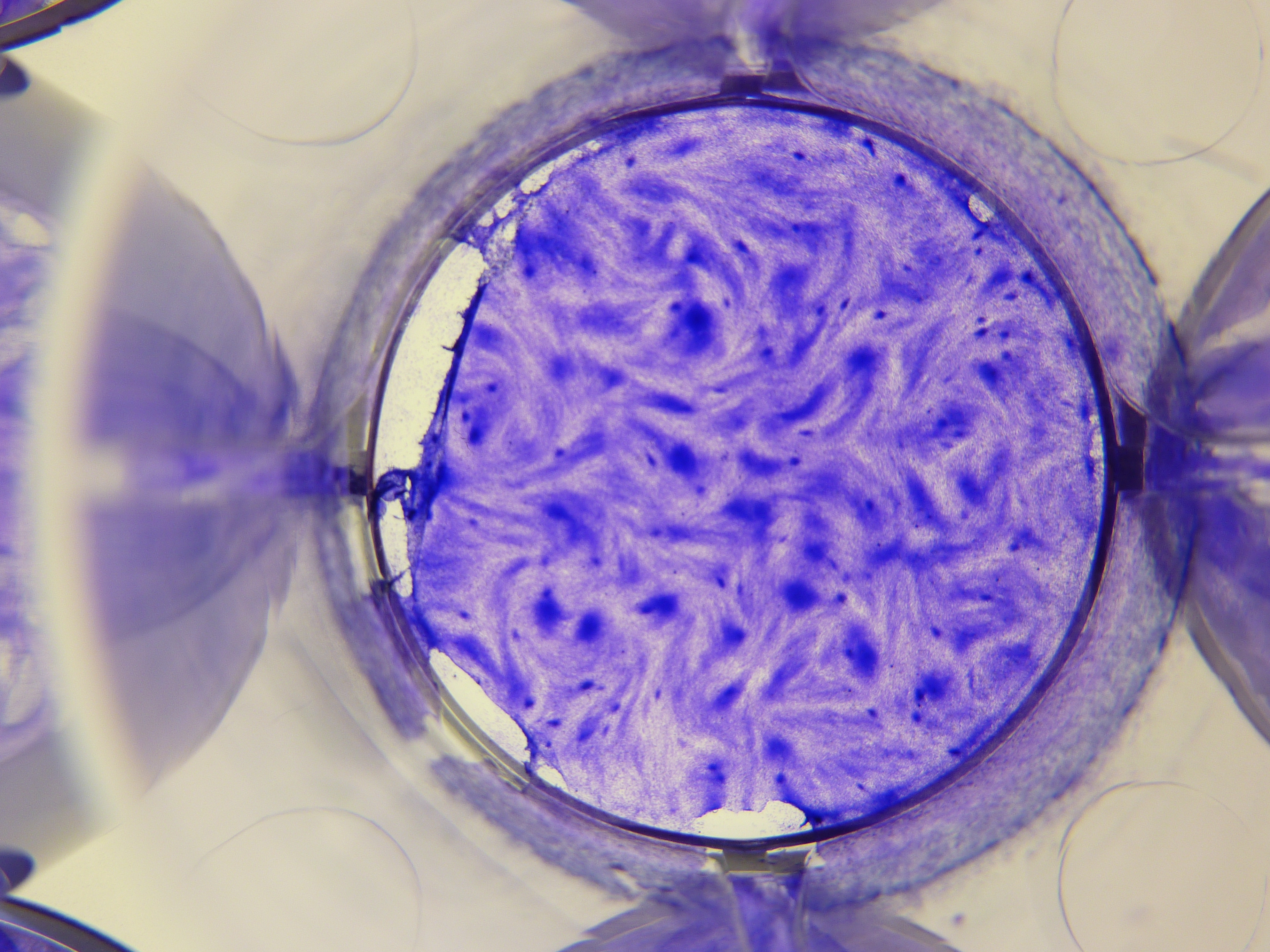

Supplement: Supplementary file 16 — Source data Fig. 7 [file 44318_2024_171_MOESM16_ESM.zip › Figure 7/7F/DCS2+IAA.JPG]

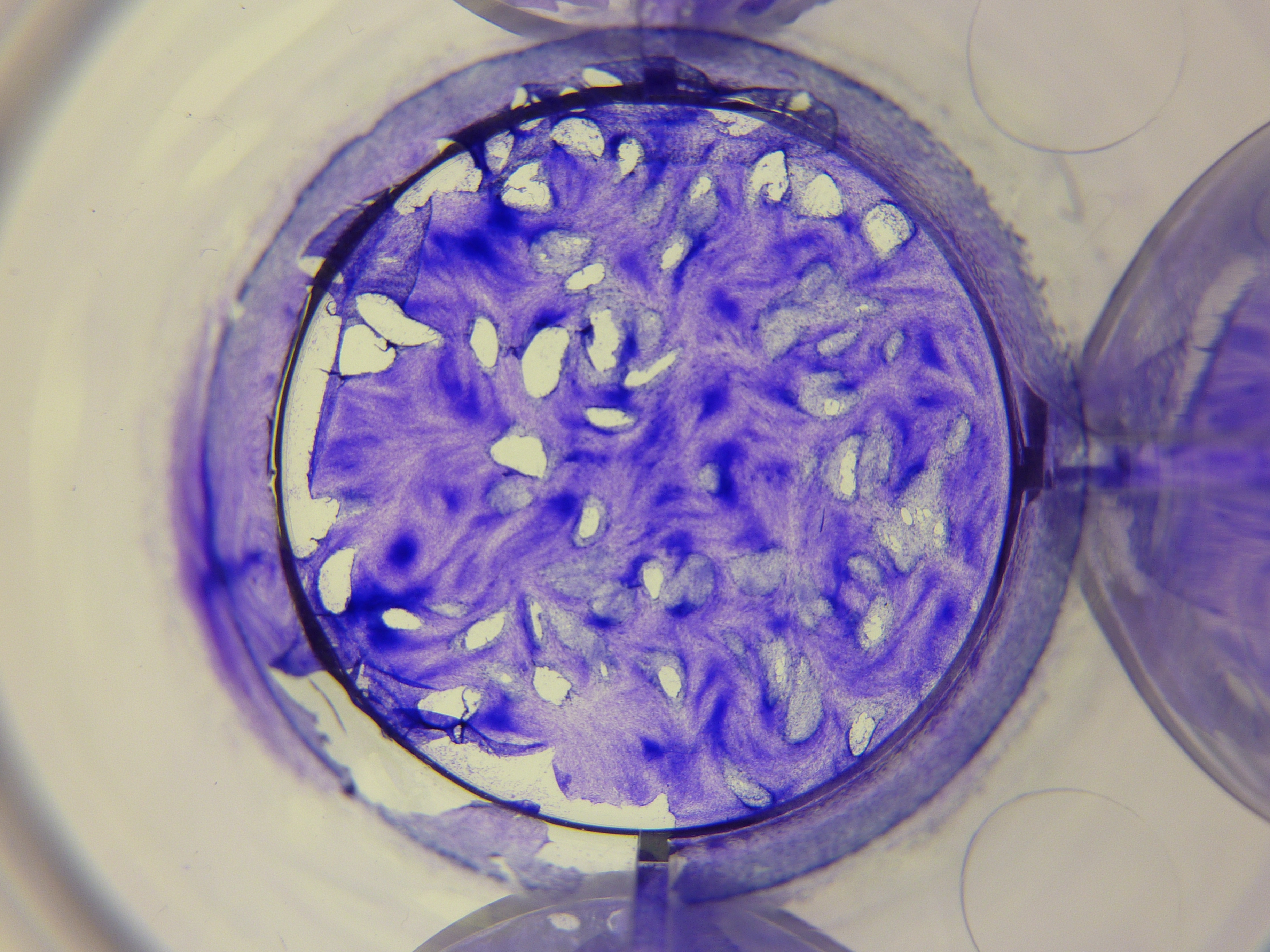

Supplement: Supplementary file 16 — Source data Fig. 7 [file 44318_2024_171_MOESM16_ESM.zip › Figure 7/7F/DCS2-IAA.JPG]

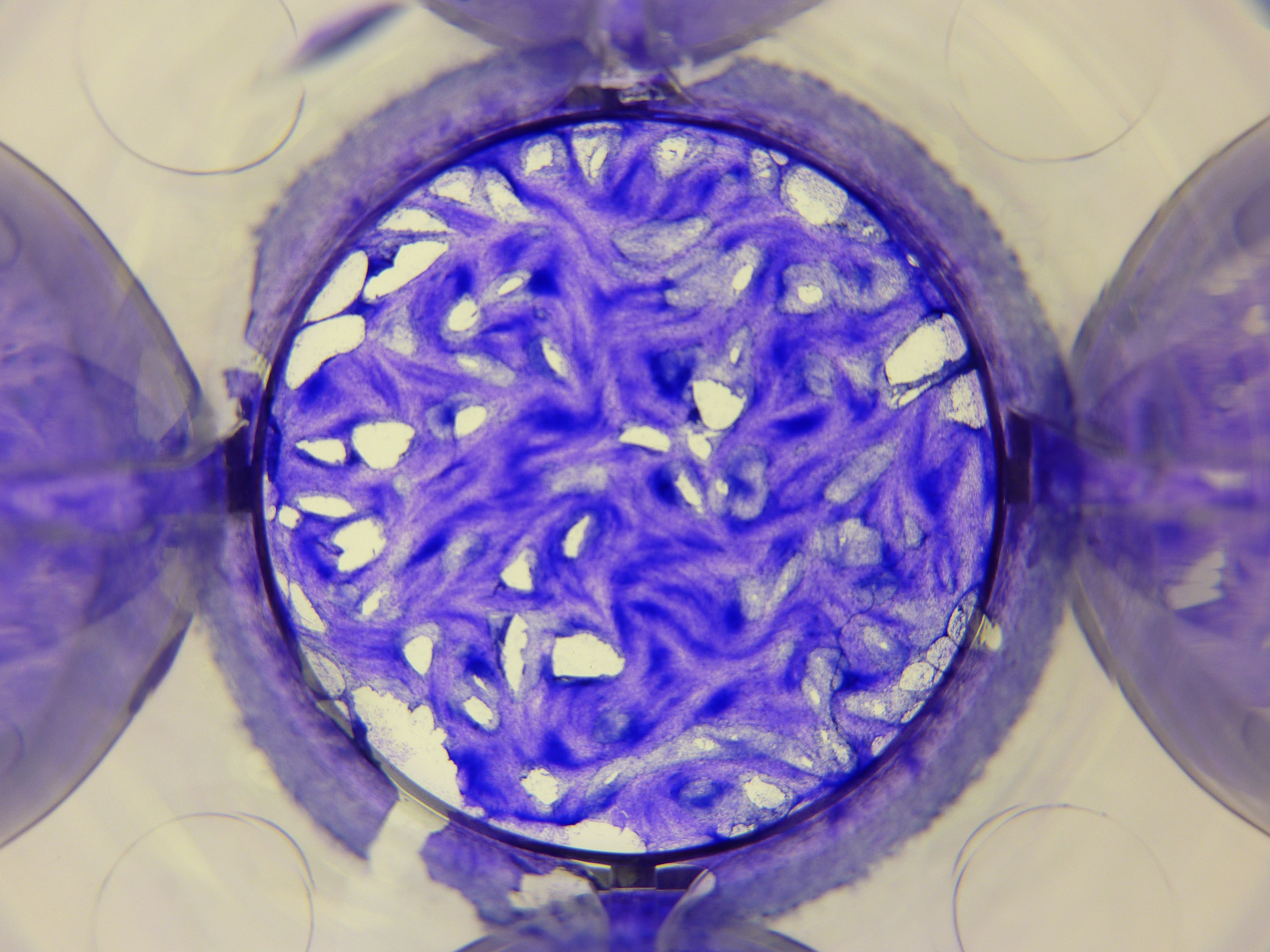

Supplement: Supplementary file 16 — Source data Fig. 7 [file 44318_2024_171_MOESM16_ESM.zip › Figure 7/7F/DCS2_24IAA_wo.JPG]

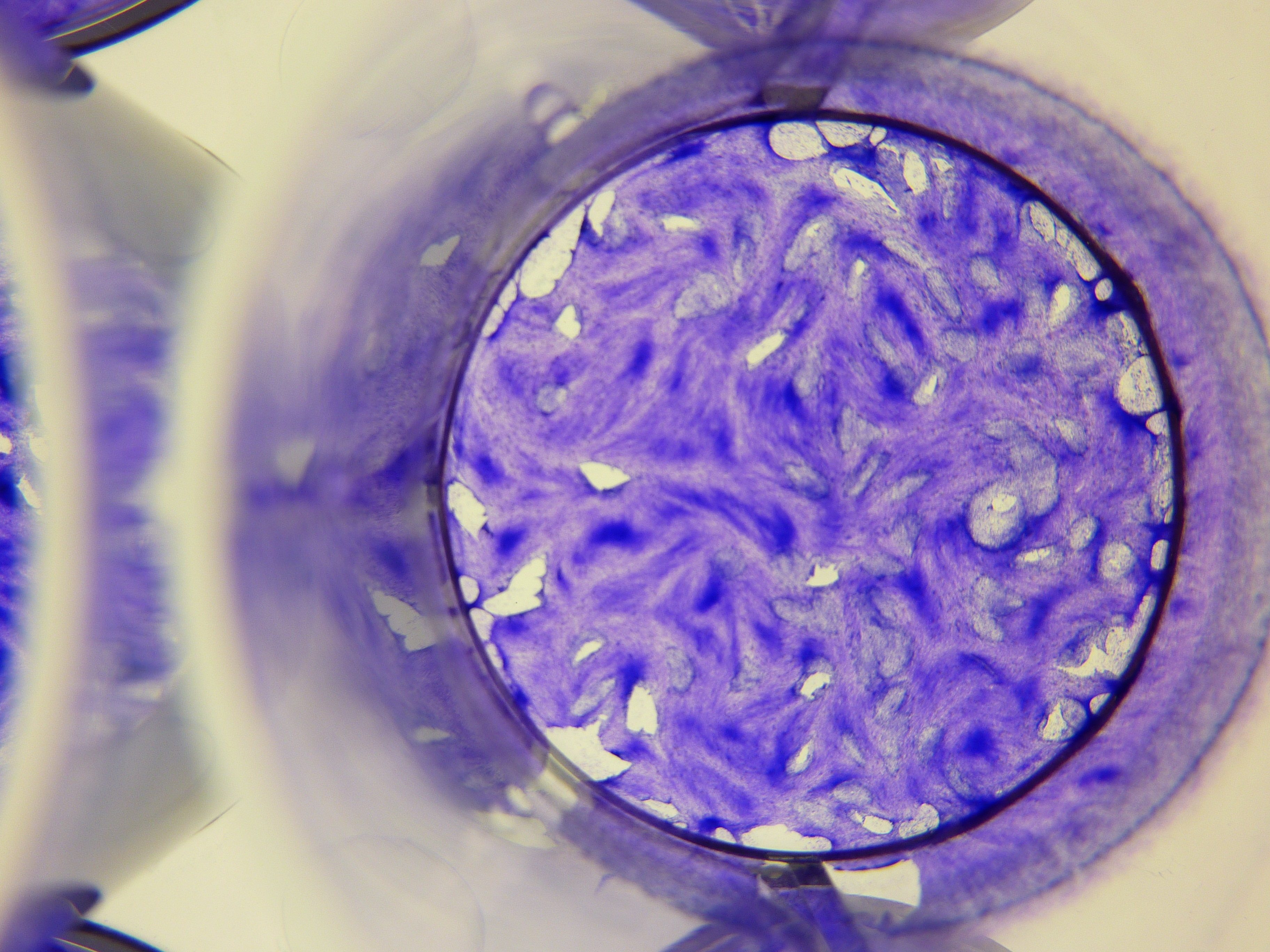

Supplement: Supplementary file 16 — Source data Fig. 7 [file 44318_2024_171_MOESM16_ESM.zip › Figure 7/7F/DCS2_48IAA_wo.JPG]

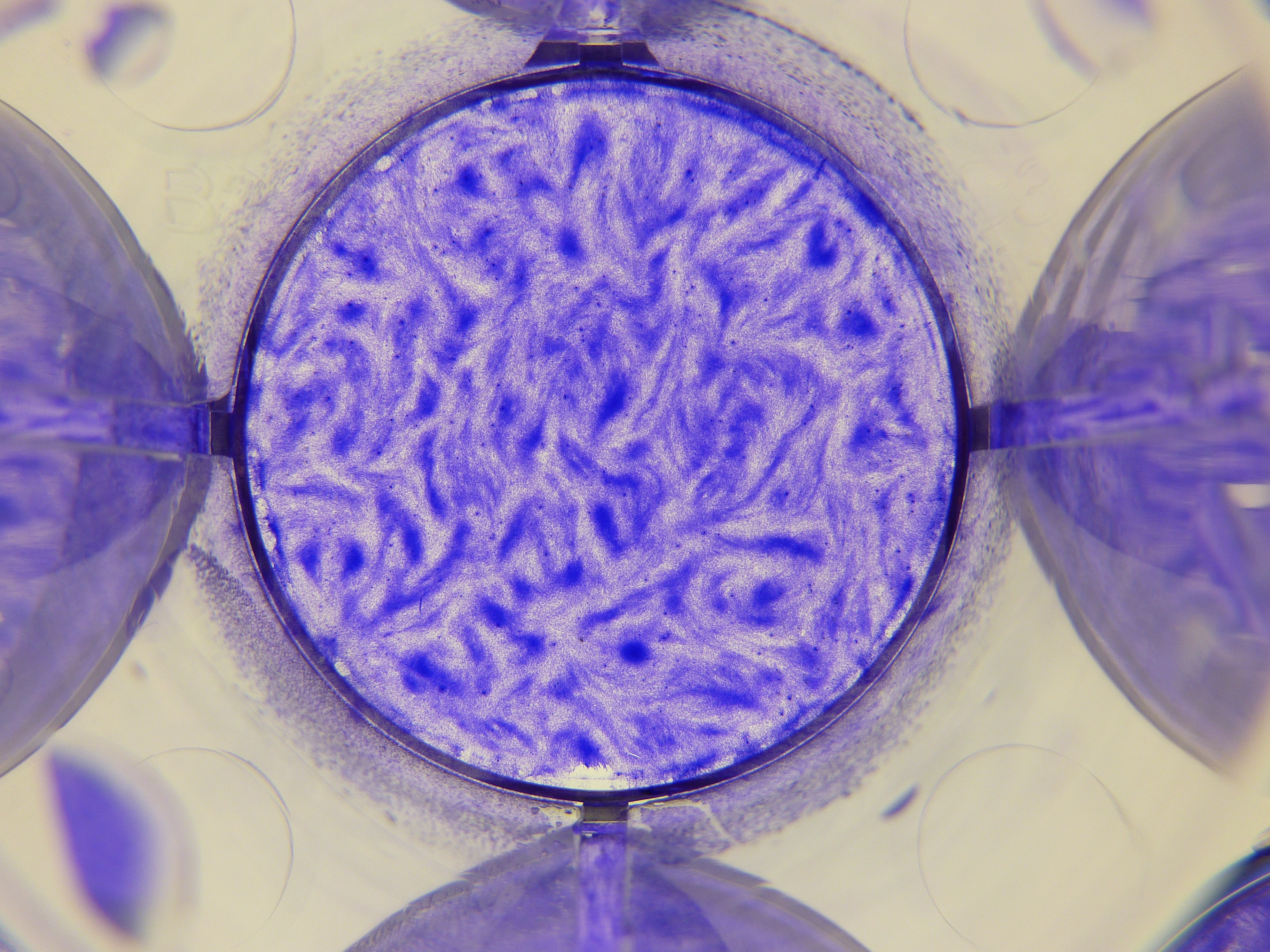

Supplement: Supplementary file 16 — Source data Fig. 7 [file 44318_2024_171_MOESM16_ESM.zip › Figure 7/7F/GAPM3+IAA.JPG]

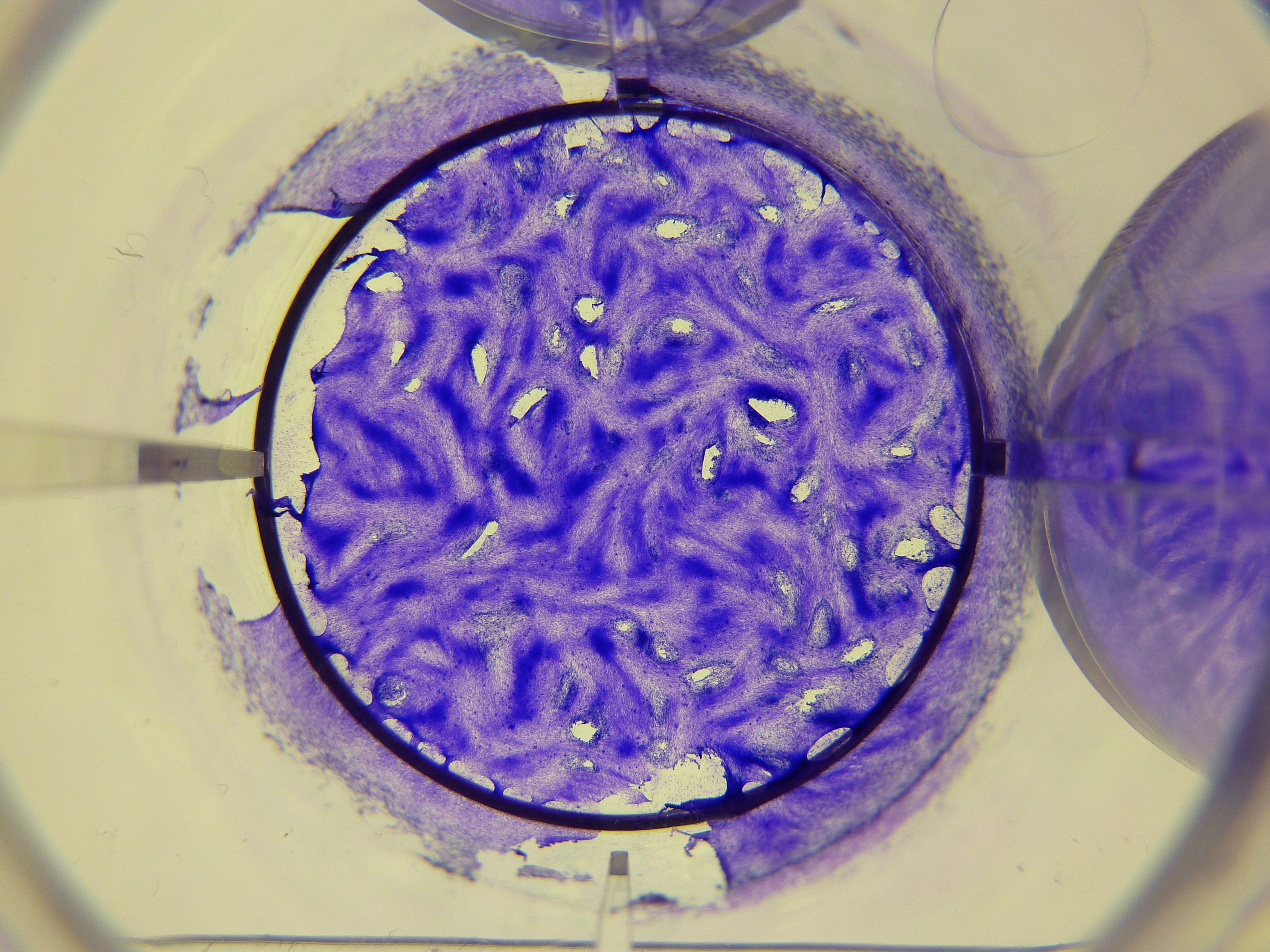

Supplement: Supplementary file 16 — Source data Fig. 7 [file 44318_2024_171_MOESM16_ESM.zip › Figure 7/7F/GAPM3-IAA.JPG]

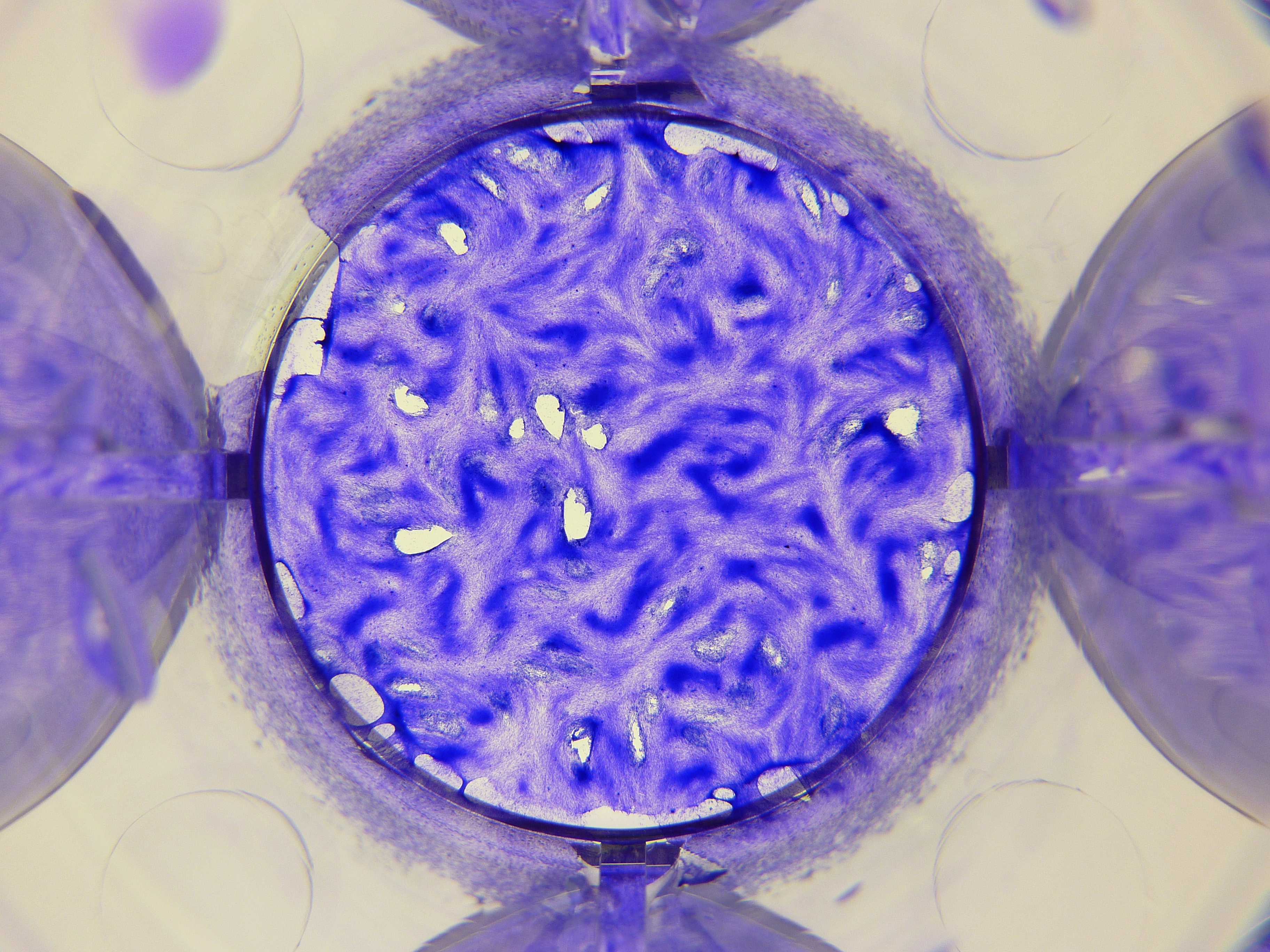

Supplement: Supplementary file 16 — Source data Fig. 7 [file 44318_2024_171_MOESM16_ESM.zip › Figure 7/7F/GAPM3_24IAA_wo.JPG]

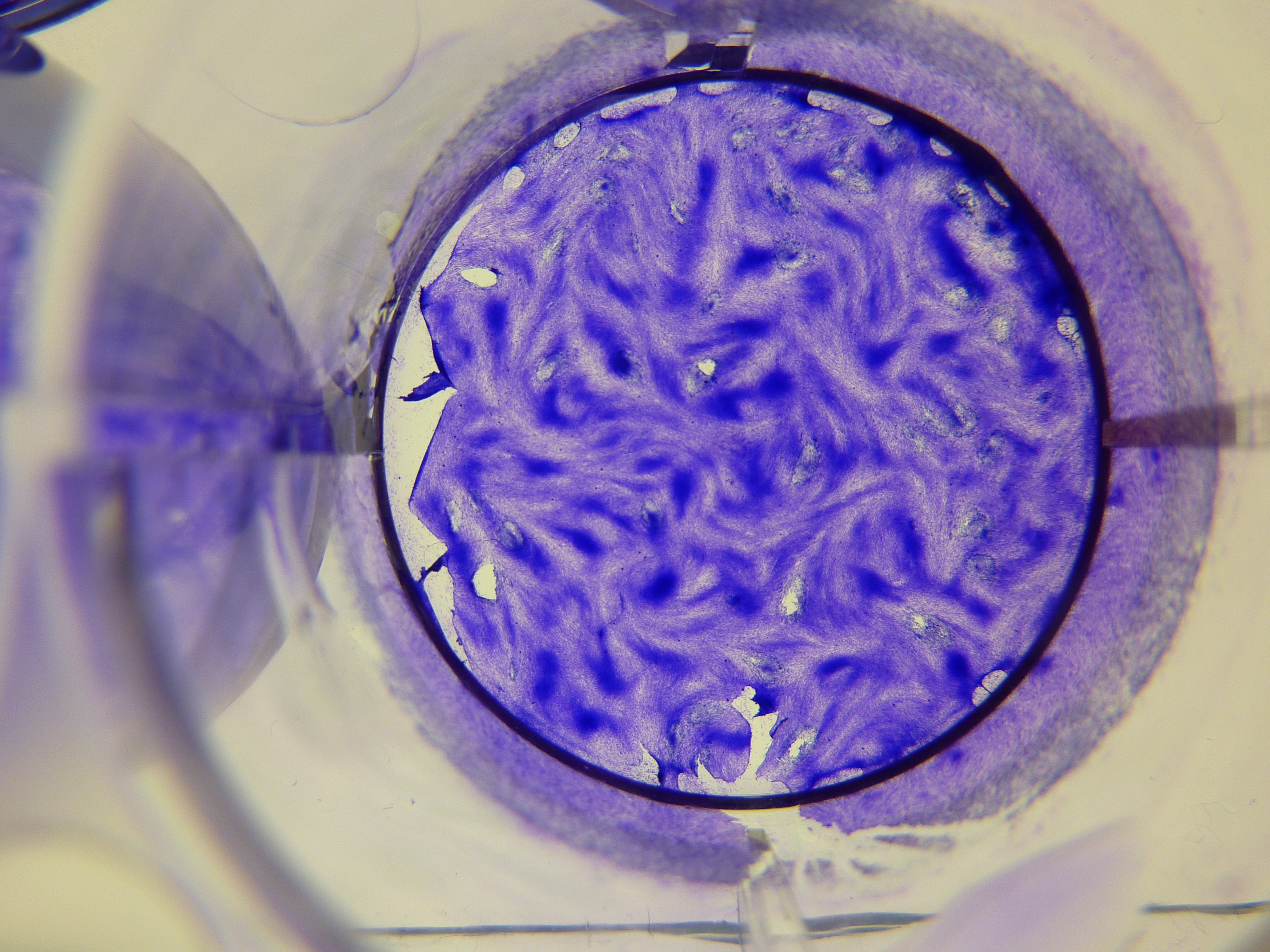

Supplement: Supplementary file 16 — Source data Fig. 7 [file 44318_2024_171_MOESM16_ESM.zip › Figure 7/7F/GAPM3_48IAA_wo.JPG]

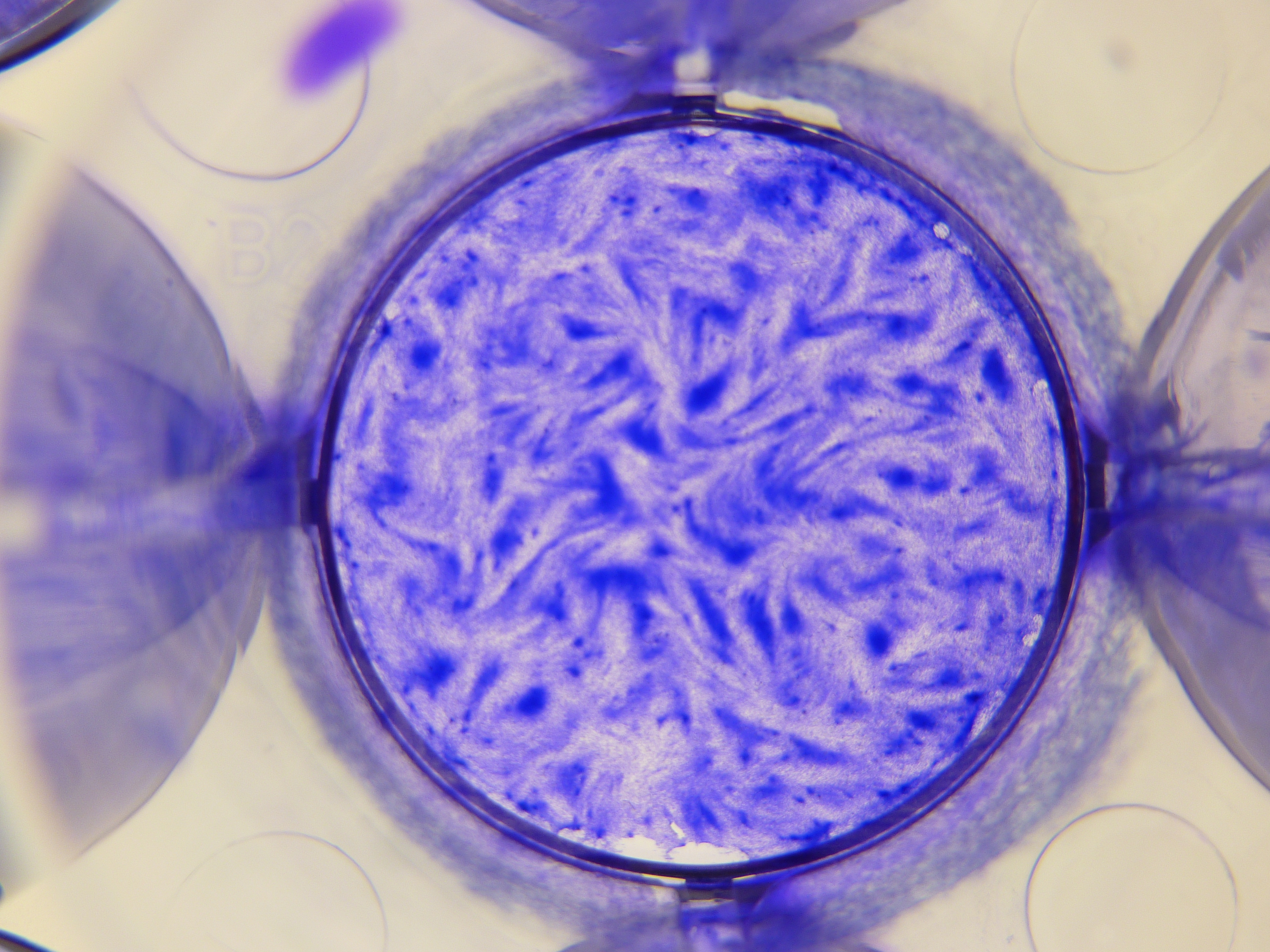

Supplement: Supplementary file 16 — Source data Fig. 7 [file 44318_2024_171_MOESM16_ESM.zip › Figure 7/7F/PP2A-B2+IAA.JPG]

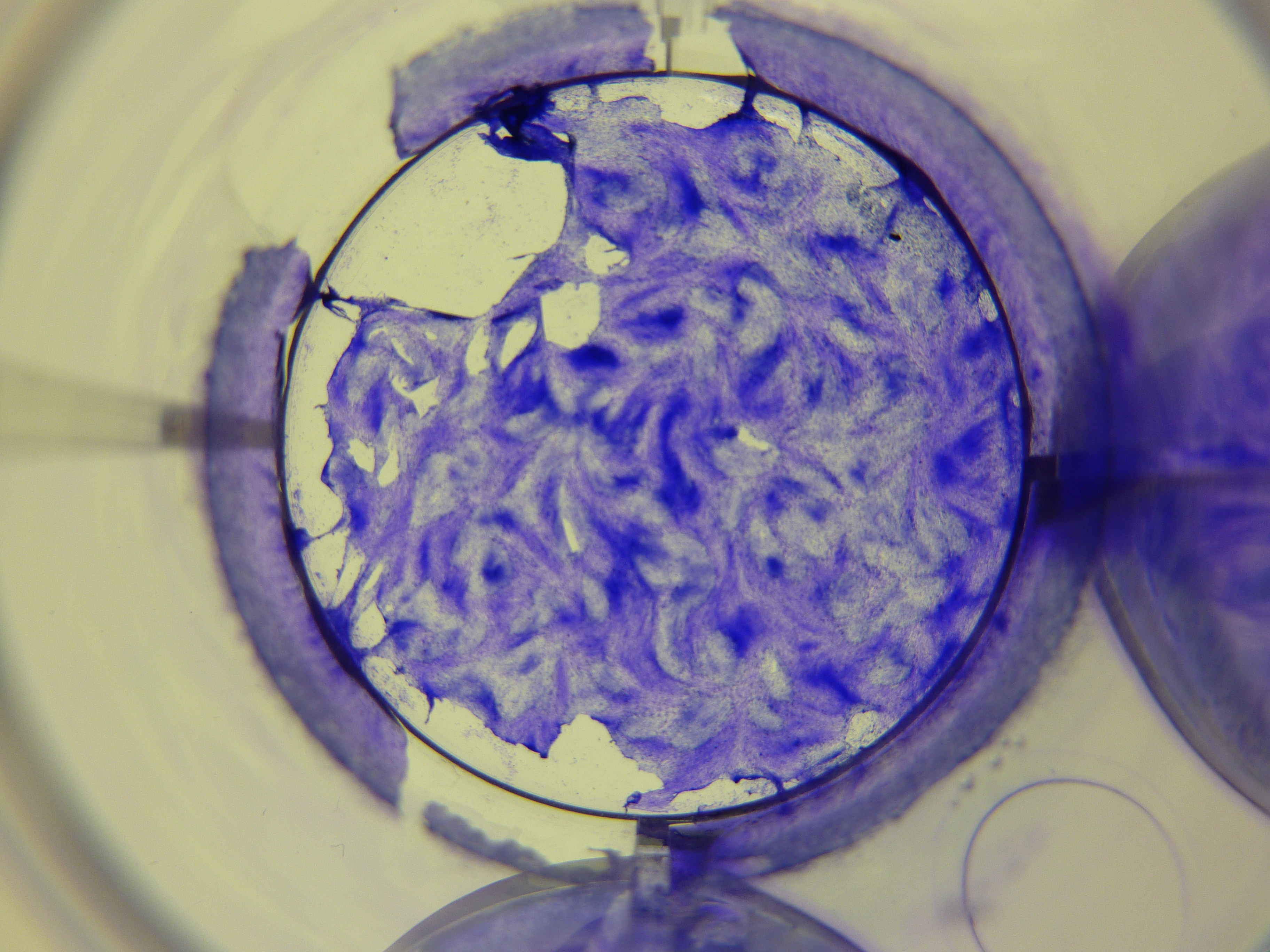

Supplement: Supplementary file 16 — Source data Fig. 7 [file 44318_2024_171_MOESM16_ESM.zip › Figure 7/7F/PP2A-B2-IAA.JPG]

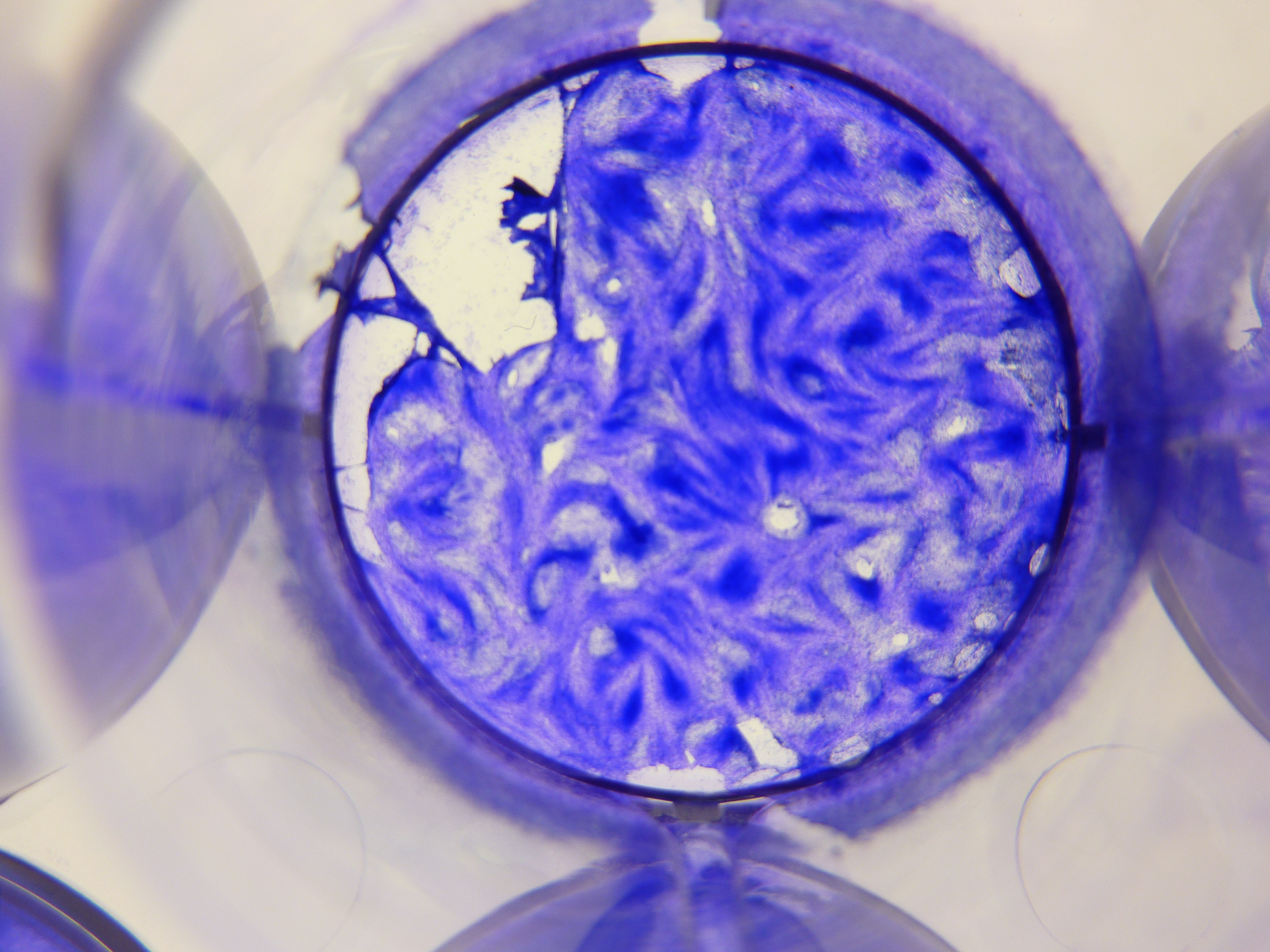

Supplement: Supplementary file 16 — Source data Fig. 7 [file 44318_2024_171_MOESM16_ESM.zip › Figure 7/7F/PP2A-B2_24IAA_wo.JPG]

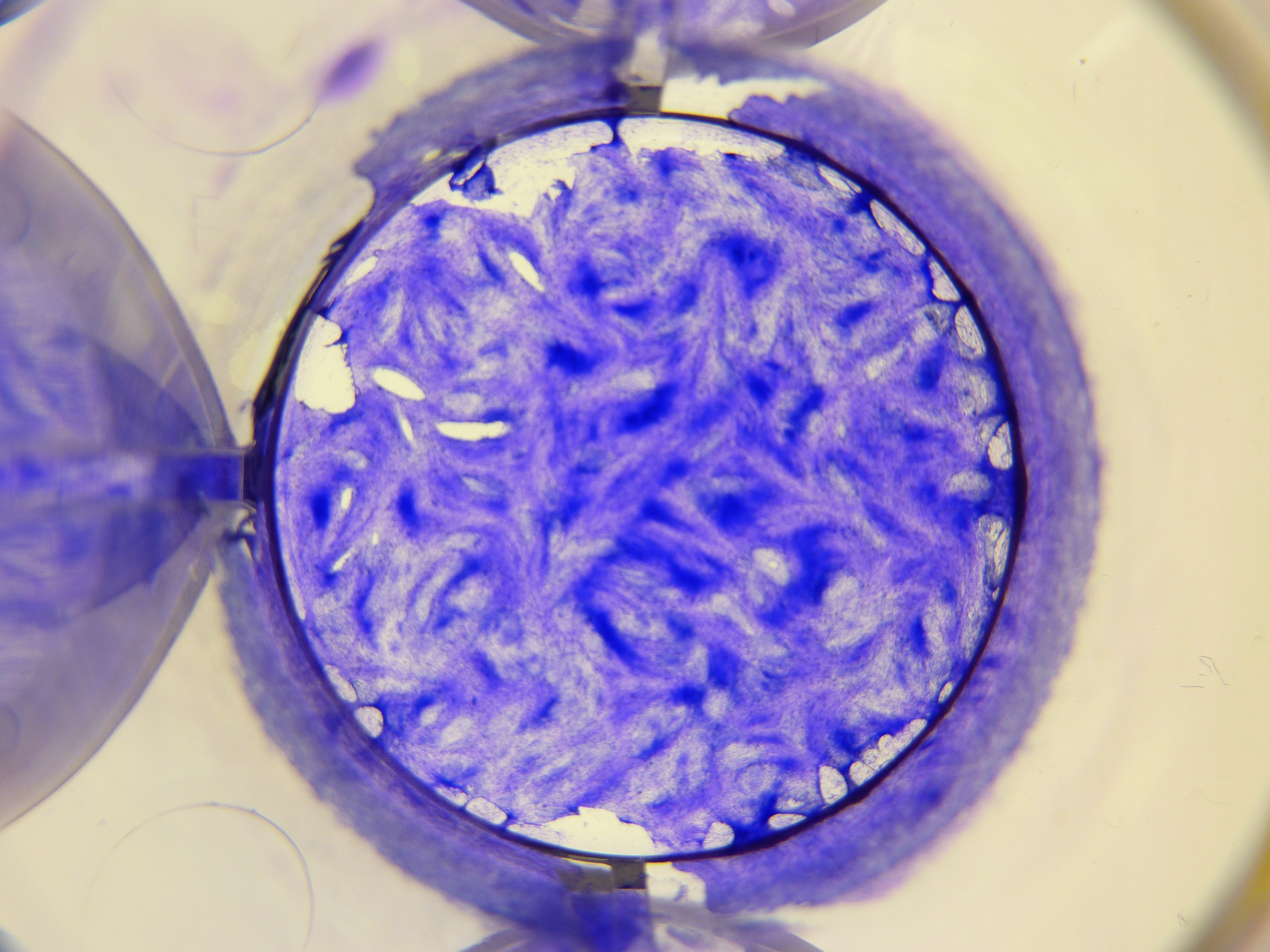

Supplement: Supplementary file 16 — Source data Fig. 7 [file 44318_2024_171_MOESM16_ESM.zip › Figure 7/7F/PP2A-B2_48IAA_wo.JPG]

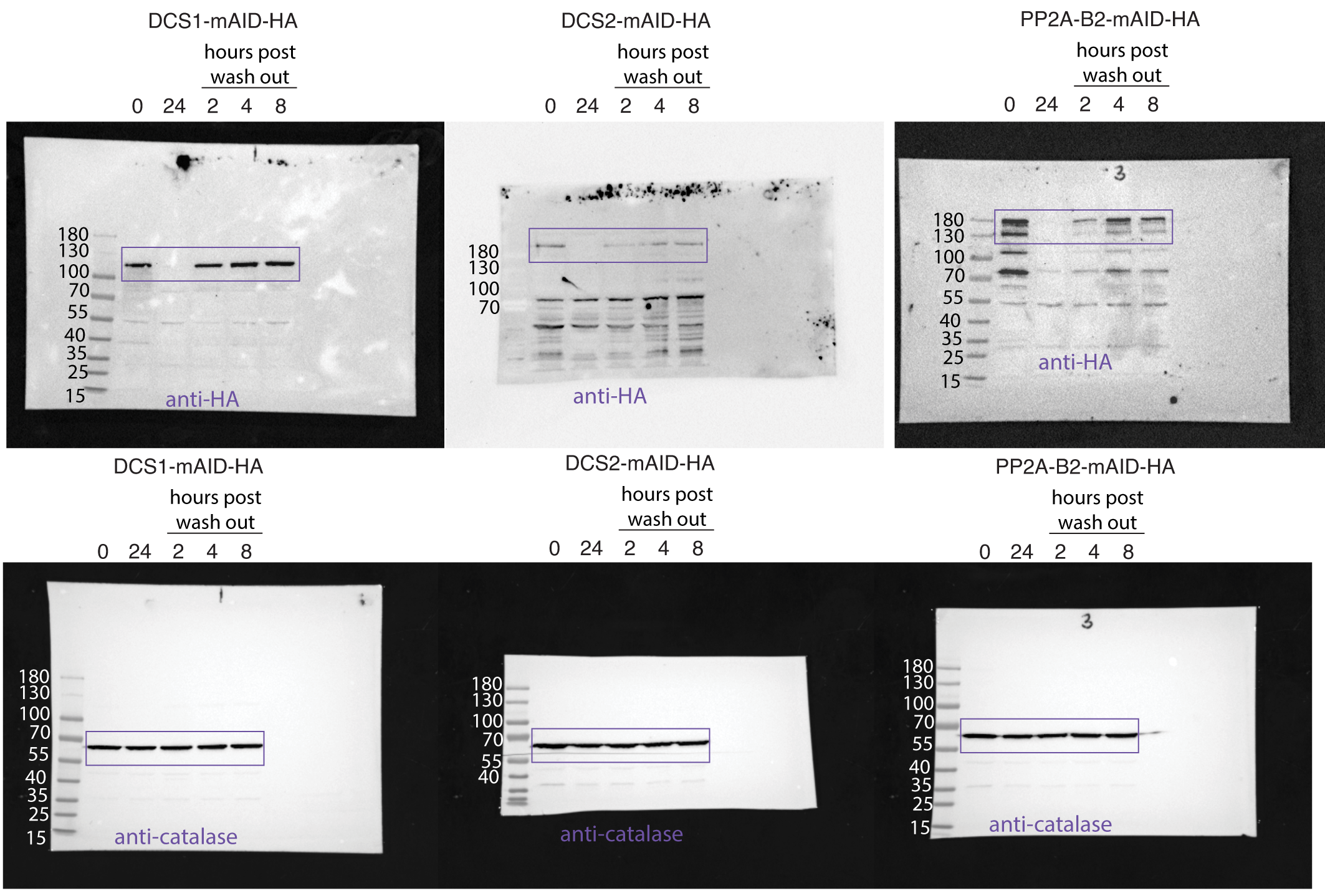

Supplement: Supplementary file 16 — Source data Fig. 7 [file 44318_2024_171_MOESM16_ESM.zip › Figure 7/7G/DCS1_DCS2_PP2A-B2_WBreversibility-01.tif]

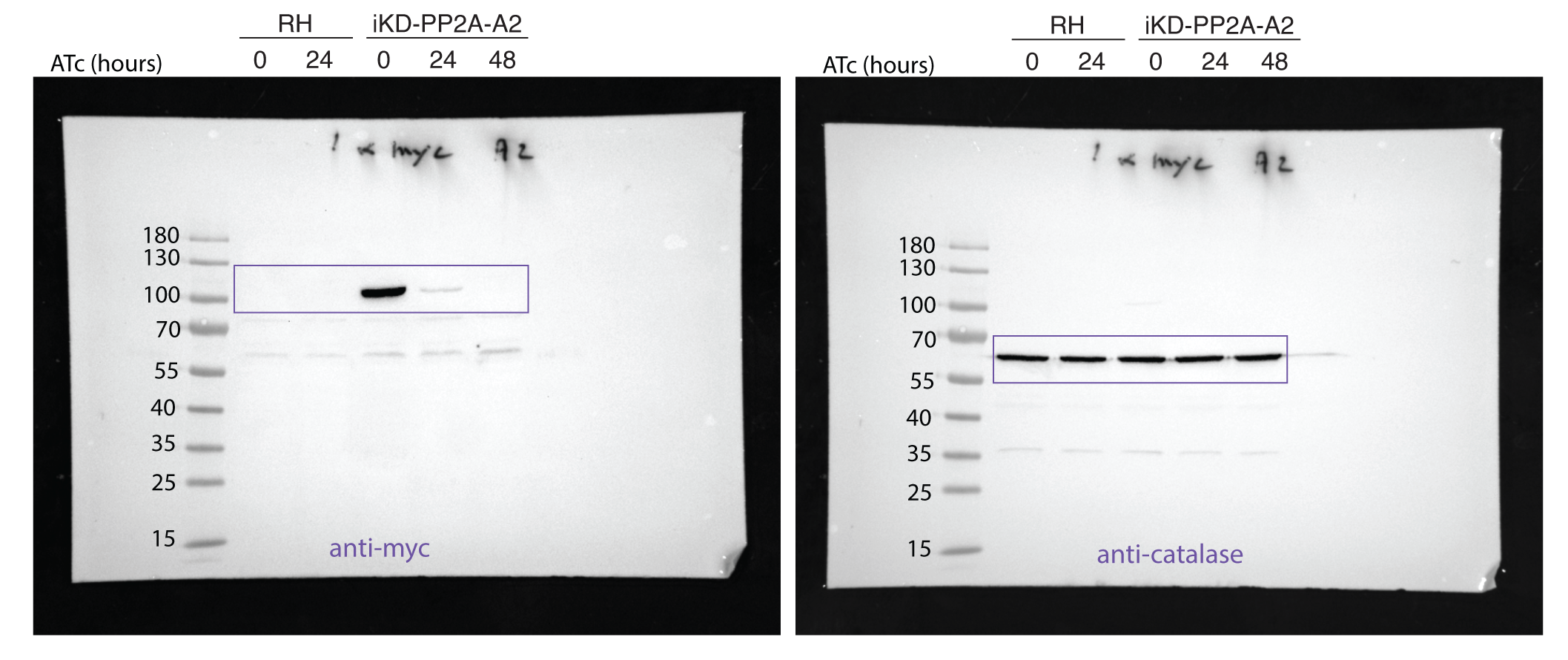

Supplement: Supplementary file 17 — Source data Fig. 9 [file 44318_2024_171_MOESM17_ESM.zip › Figure 9/9C/PP2A-A2_downregulation_myc_catalase.tif]

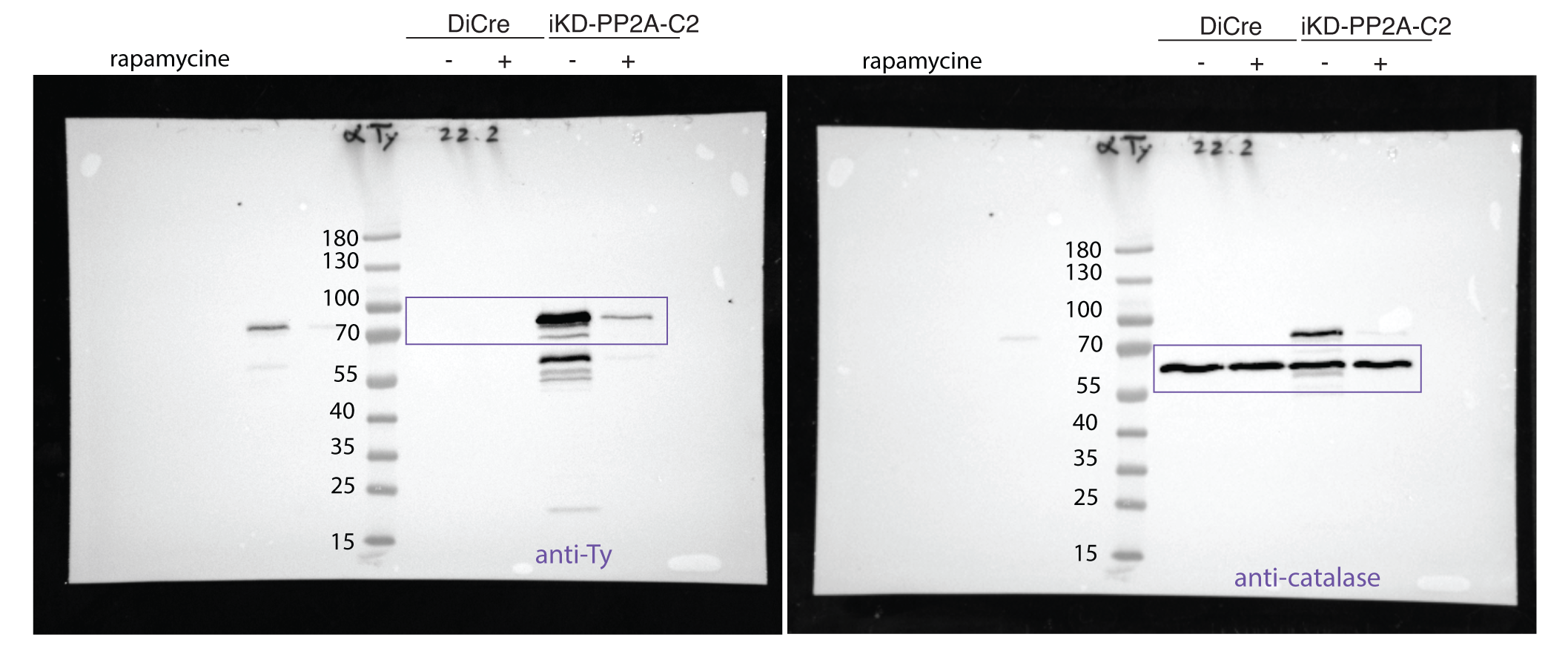

Supplement: Supplementary file 17 — Source data Fig. 9 [file 44318_2024_171_MOESM17_ESM.zip › Figure 9/9D/PP2A-C2_downregulation_Ty_catalase.tif]

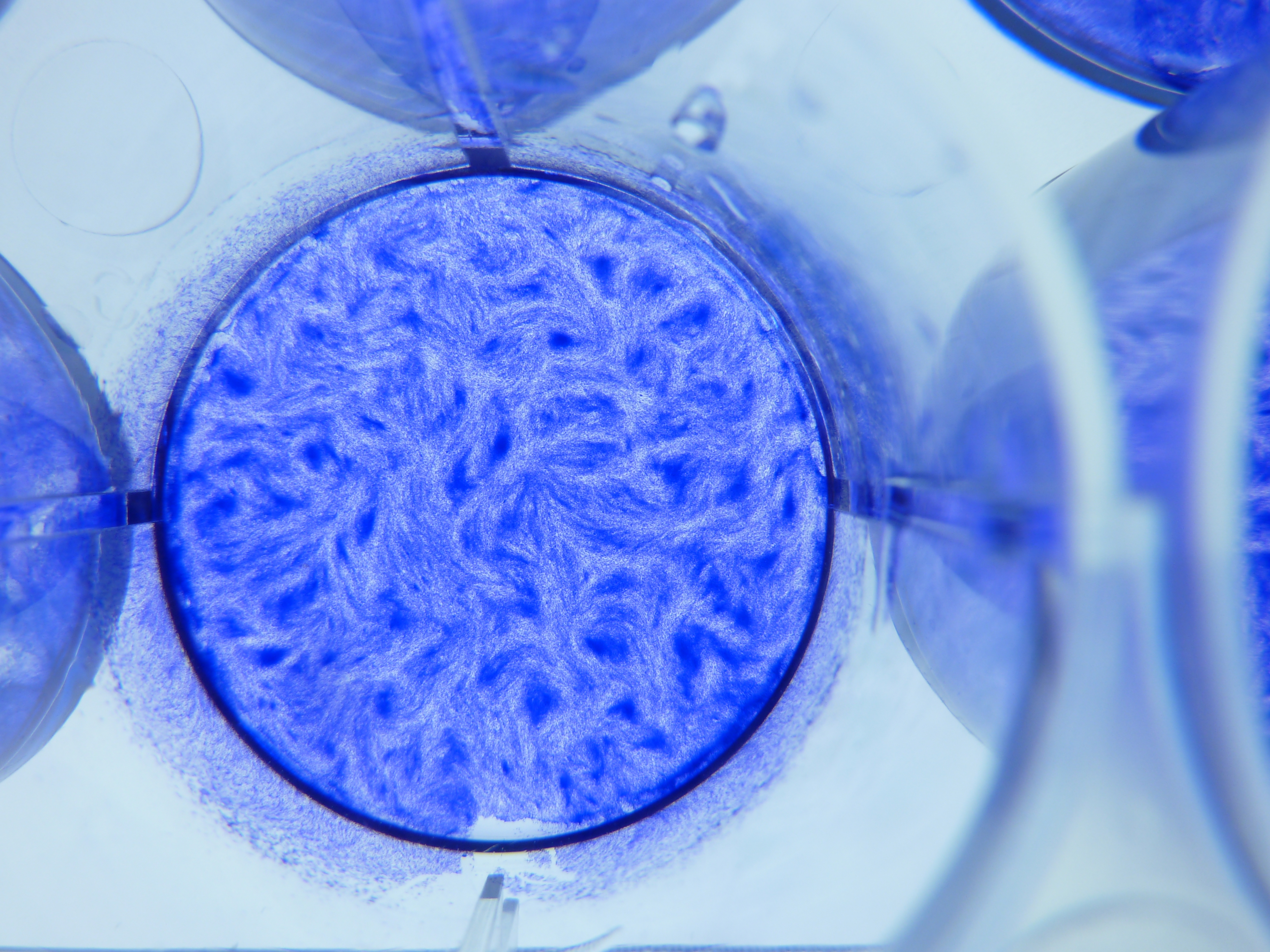

Supplement: Supplementary file 17 — Source data Fig. 9 [file 44318_2024_171_MOESM17_ESM.zip › Figure 9/9E/iKD-PP2A-A2_+ATc.JPG]

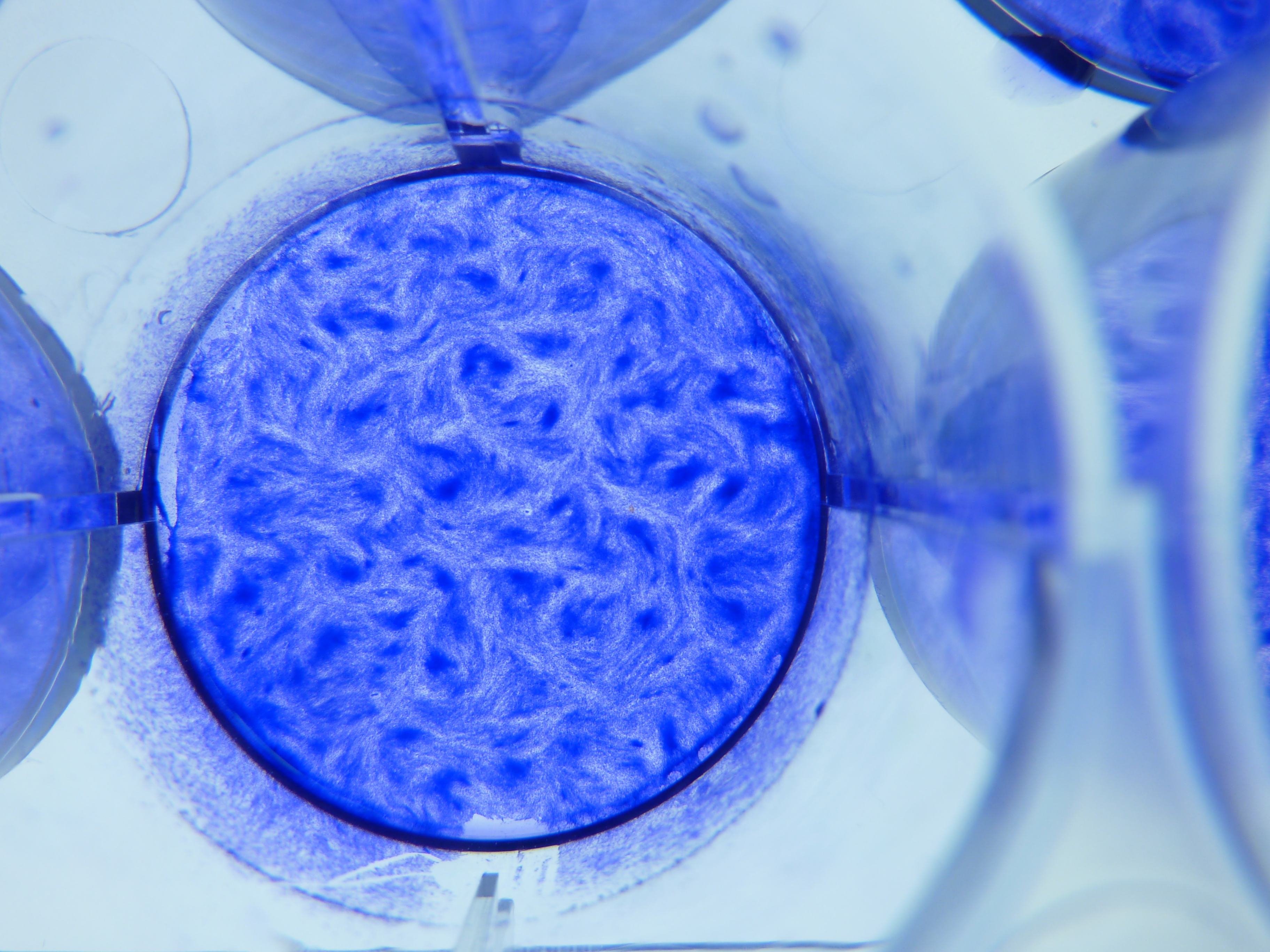

Supplement: Supplementary file 17 — Source data Fig. 9 [file 44318_2024_171_MOESM17_ESM.zip › Figure 9/9E/iKD-PP2A-A2_+ATc_24h.JPG]

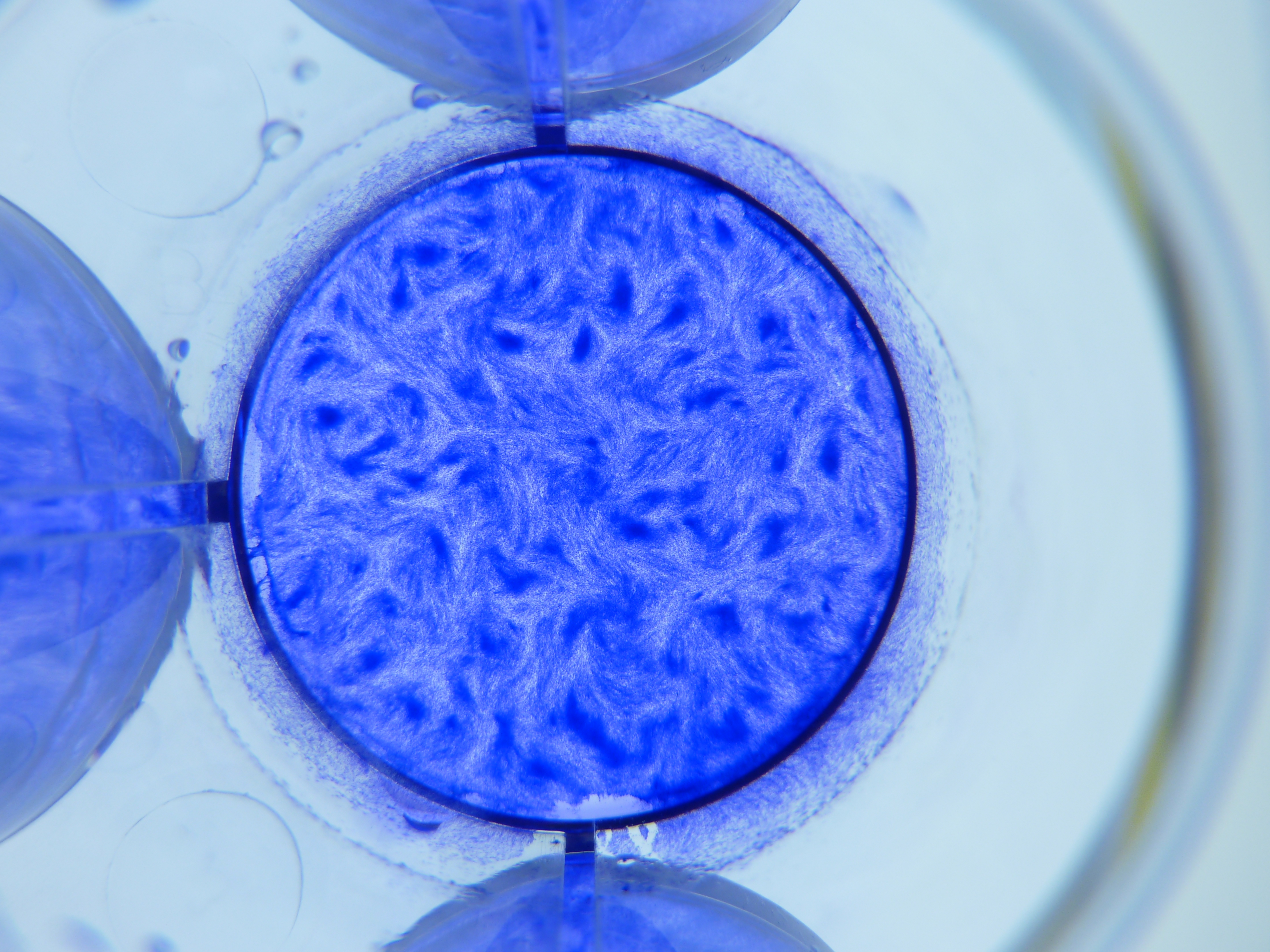

Supplement: Supplementary file 17 — Source data Fig. 9 [file 44318_2024_171_MOESM17_ESM.zip › Figure 9/9E/iKD-PP2A-A2_+ATc_48h.JPG]

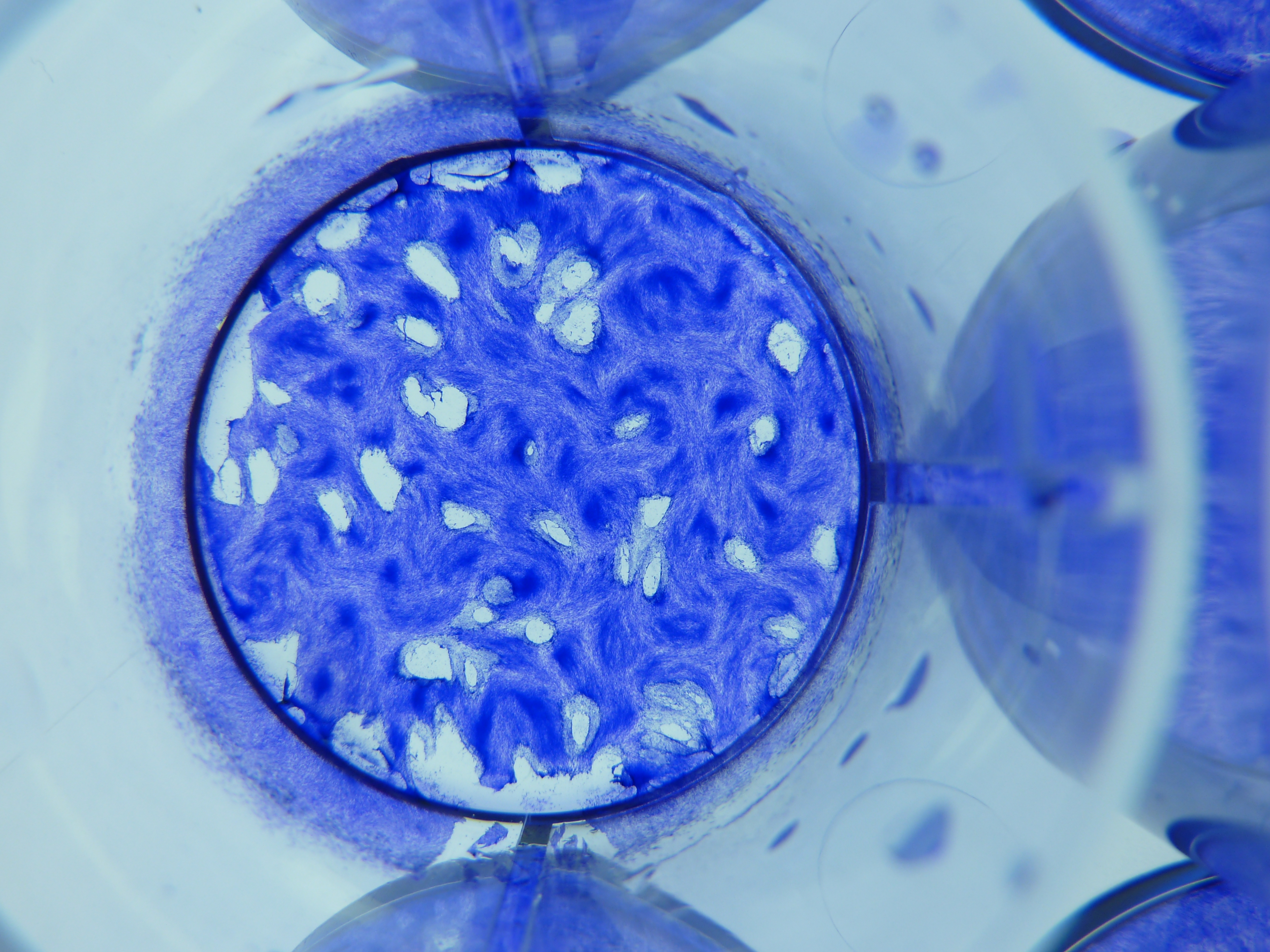

Supplement: Supplementary file 17 — Source data Fig. 9 [file 44318_2024_171_MOESM17_ESM.zip › Figure 9/9E/iKD-PP2A-A2_-ATc.JPG]

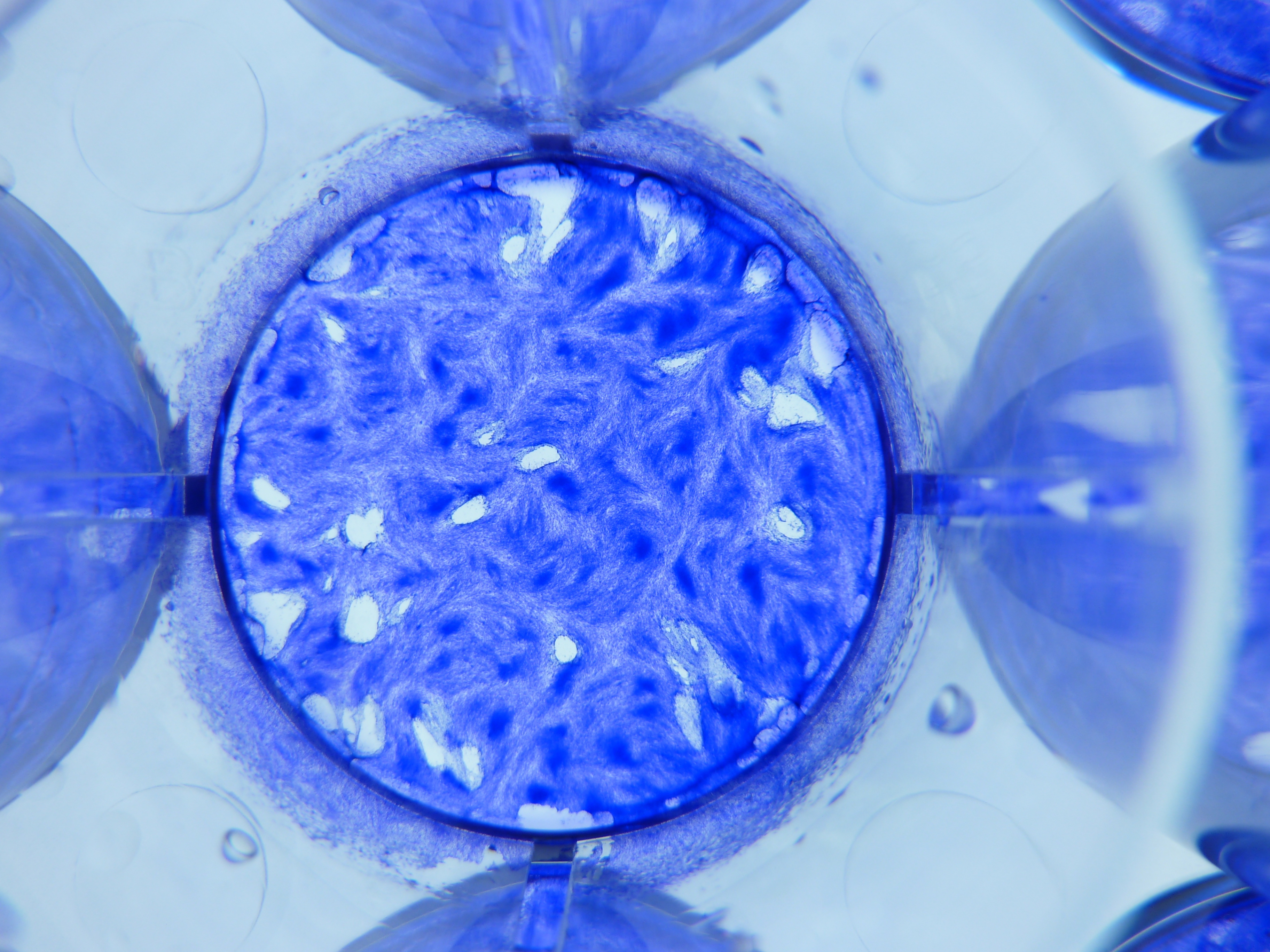

Supplement: Supplementary file 17 — Source data Fig. 9 [file 44318_2024_171_MOESM17_ESM.zip › Figure 9/9E/RH_+ATc.JPG]

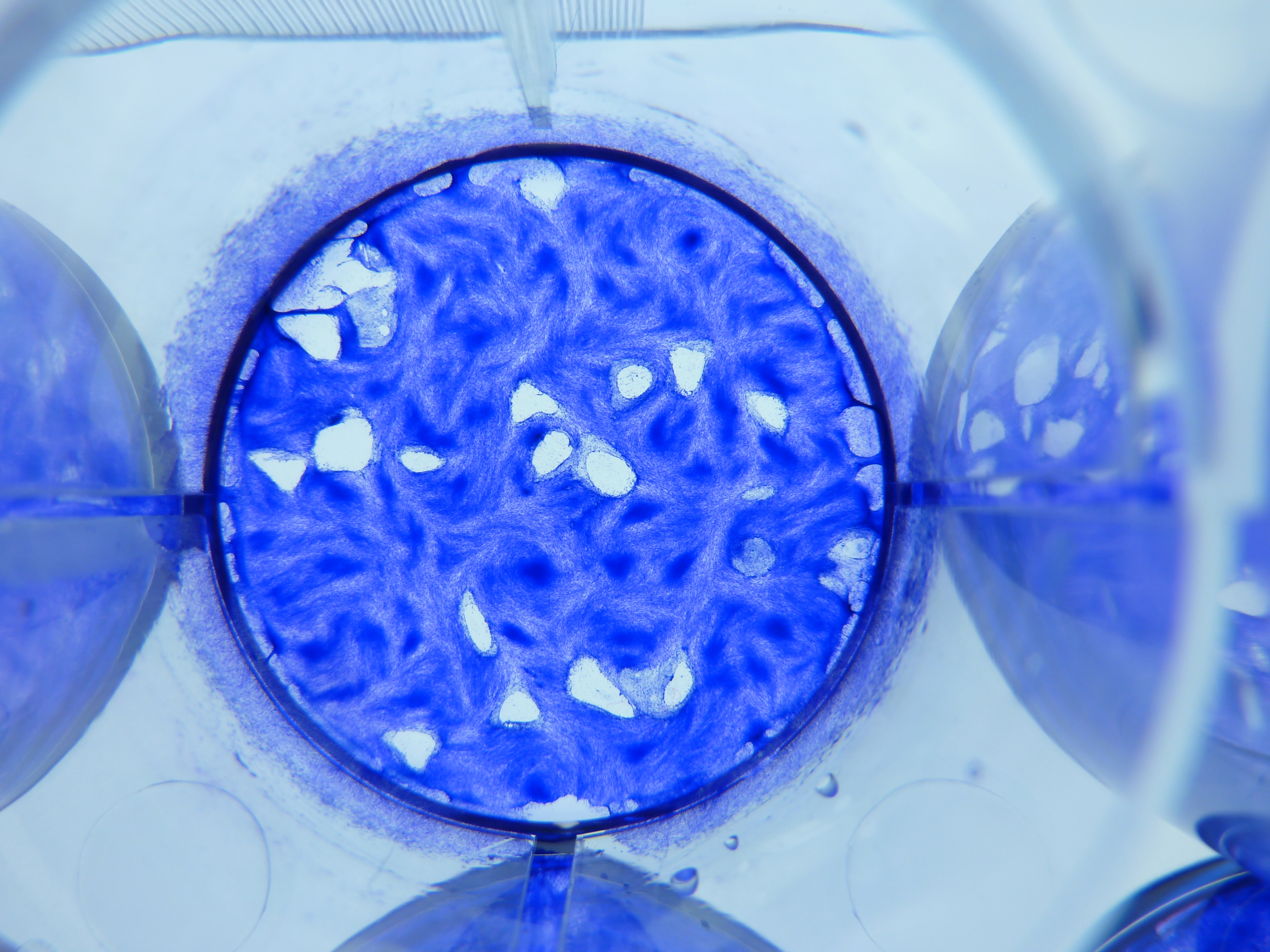

Supplement: Supplementary file 17 — Source data Fig. 9 [file 44318_2024_171_MOESM17_ESM.zip › Figure 9/9E/RH_+ATc_24h.JPG]

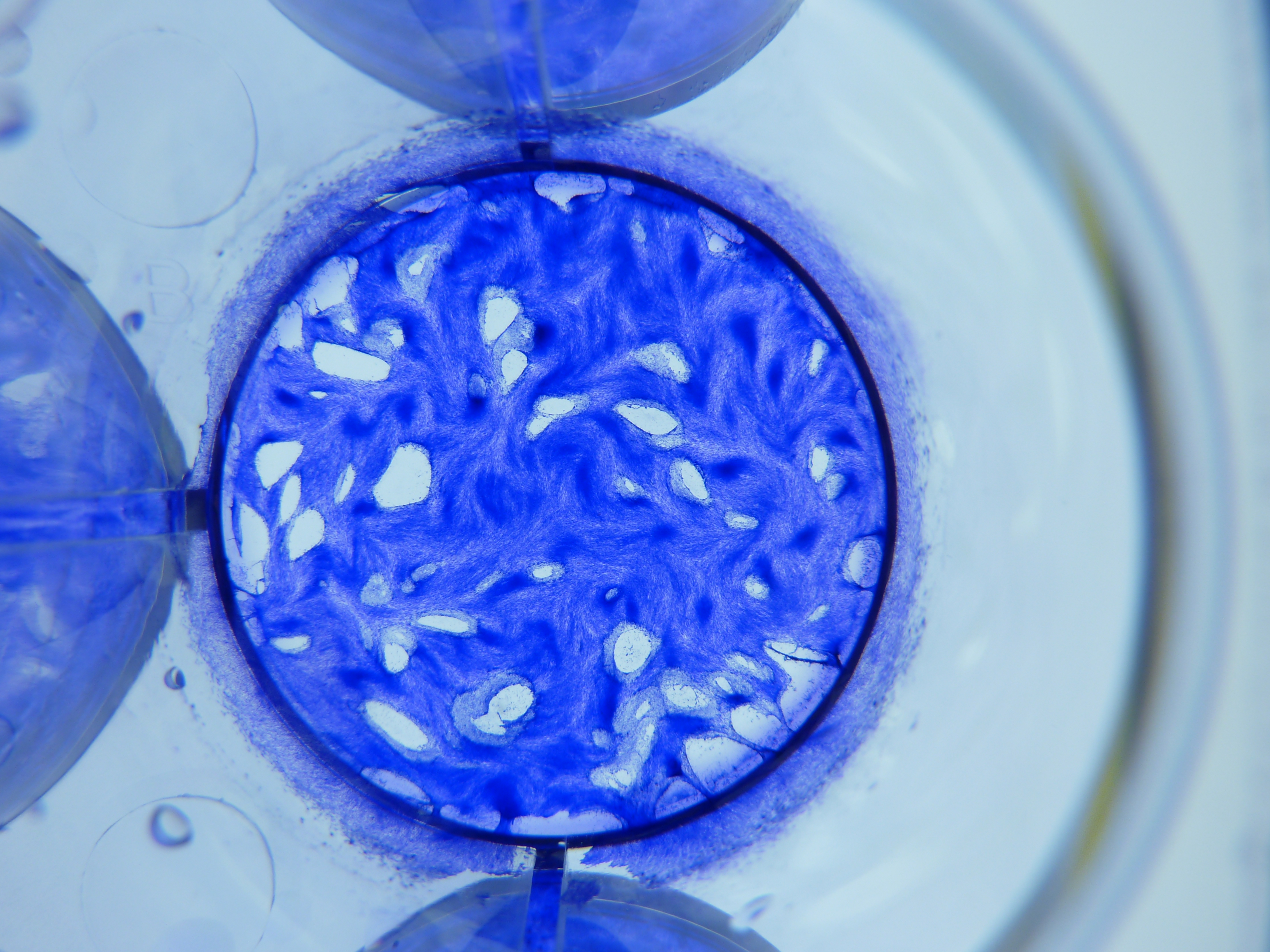

Supplement: Supplementary file 17 — Source data Fig. 9 [file 44318_2024_171_MOESM17_ESM.zip › Figure 9/9E/RH_+ATc_48h.JPG]

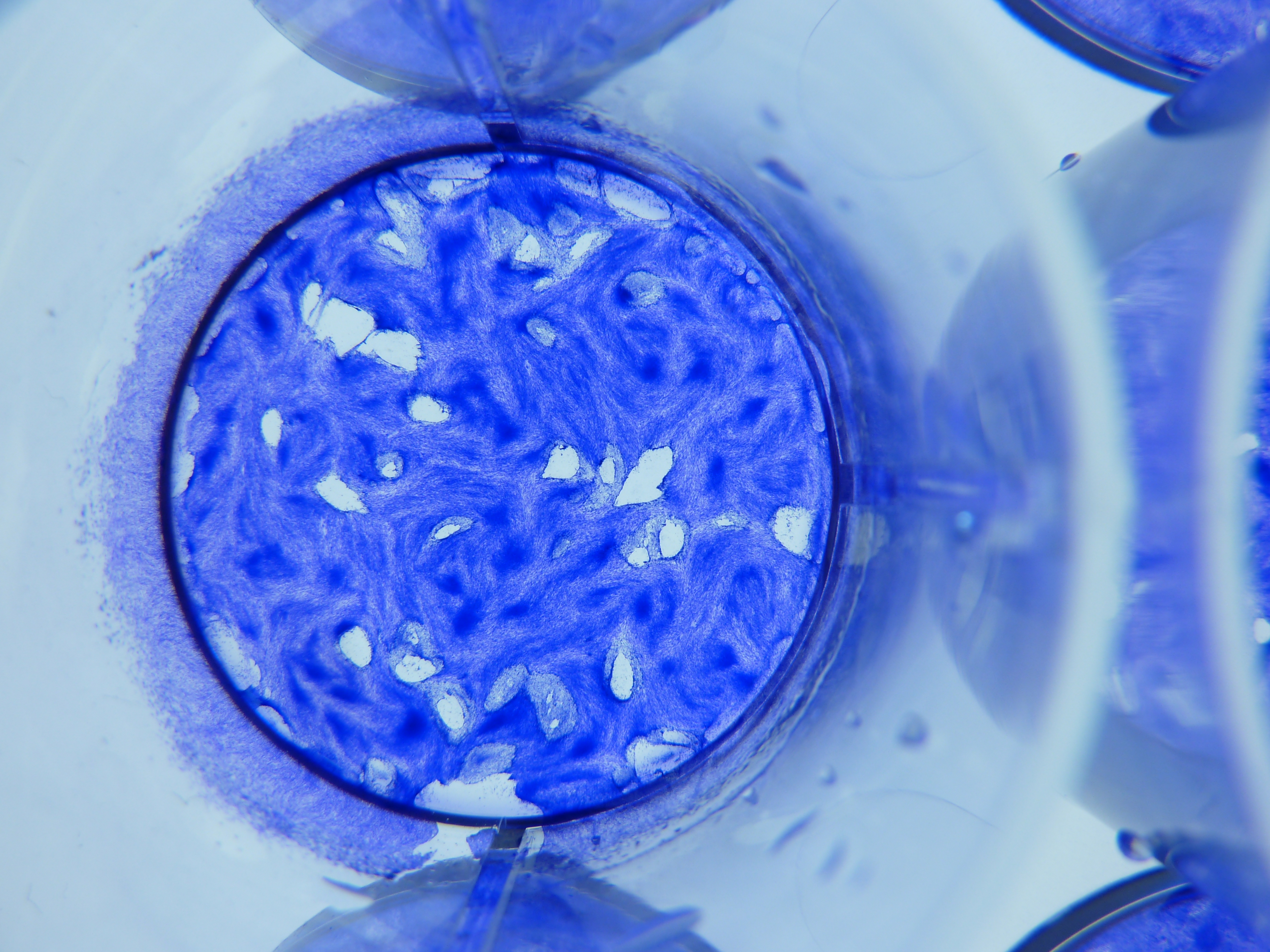

Supplement: Supplementary file 17 — Source data Fig. 9 [file 44318_2024_171_MOESM17_ESM.zip › Figure 9/9E/RH_-ATc.JPG]

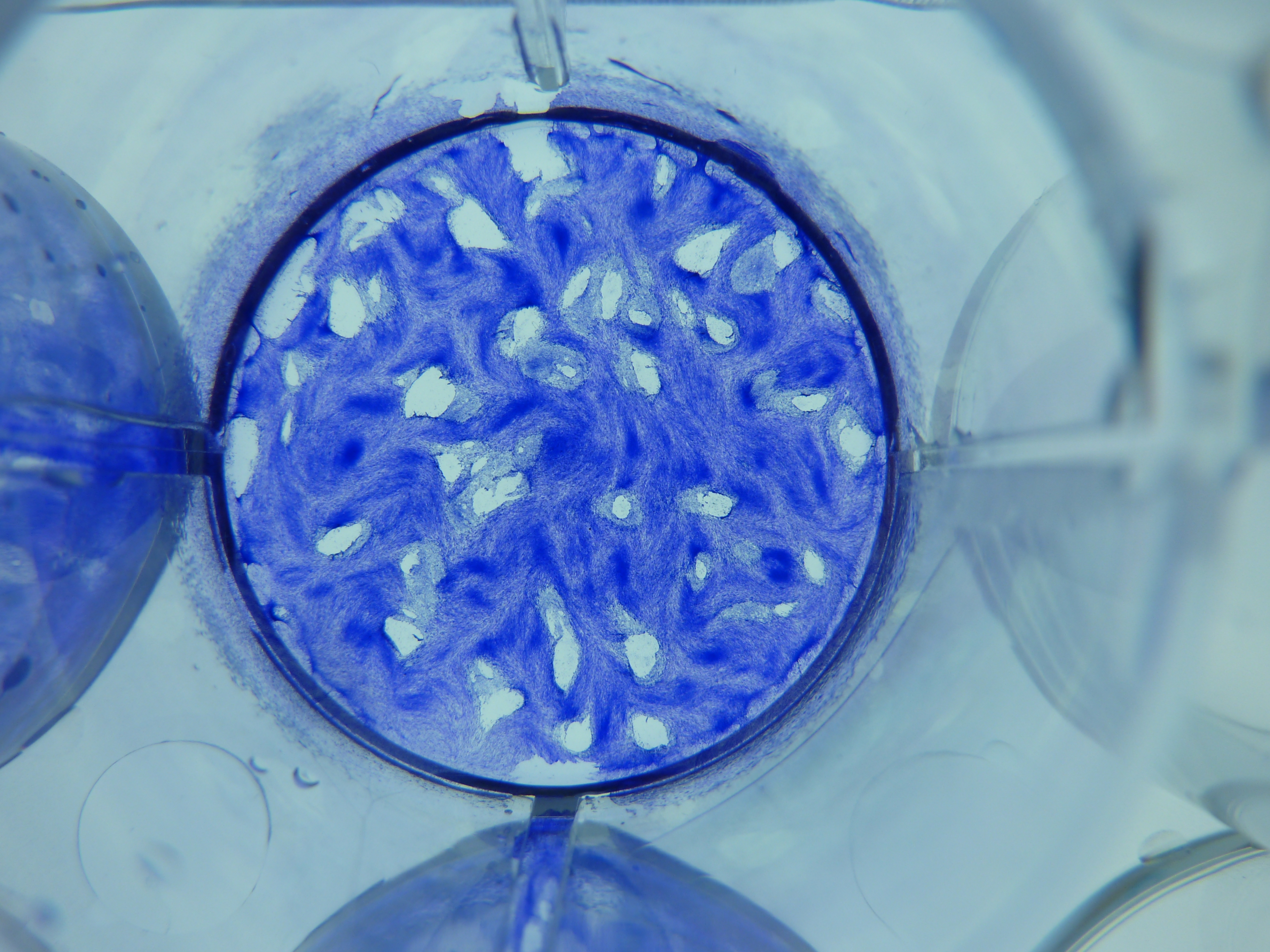

Supplement: Supplementary file 17 — Source data Fig. 9 [file 44318_2024_171_MOESM17_ESM.zip › Figure 9/9F/PA_DiCre_+Rapa.JPG]

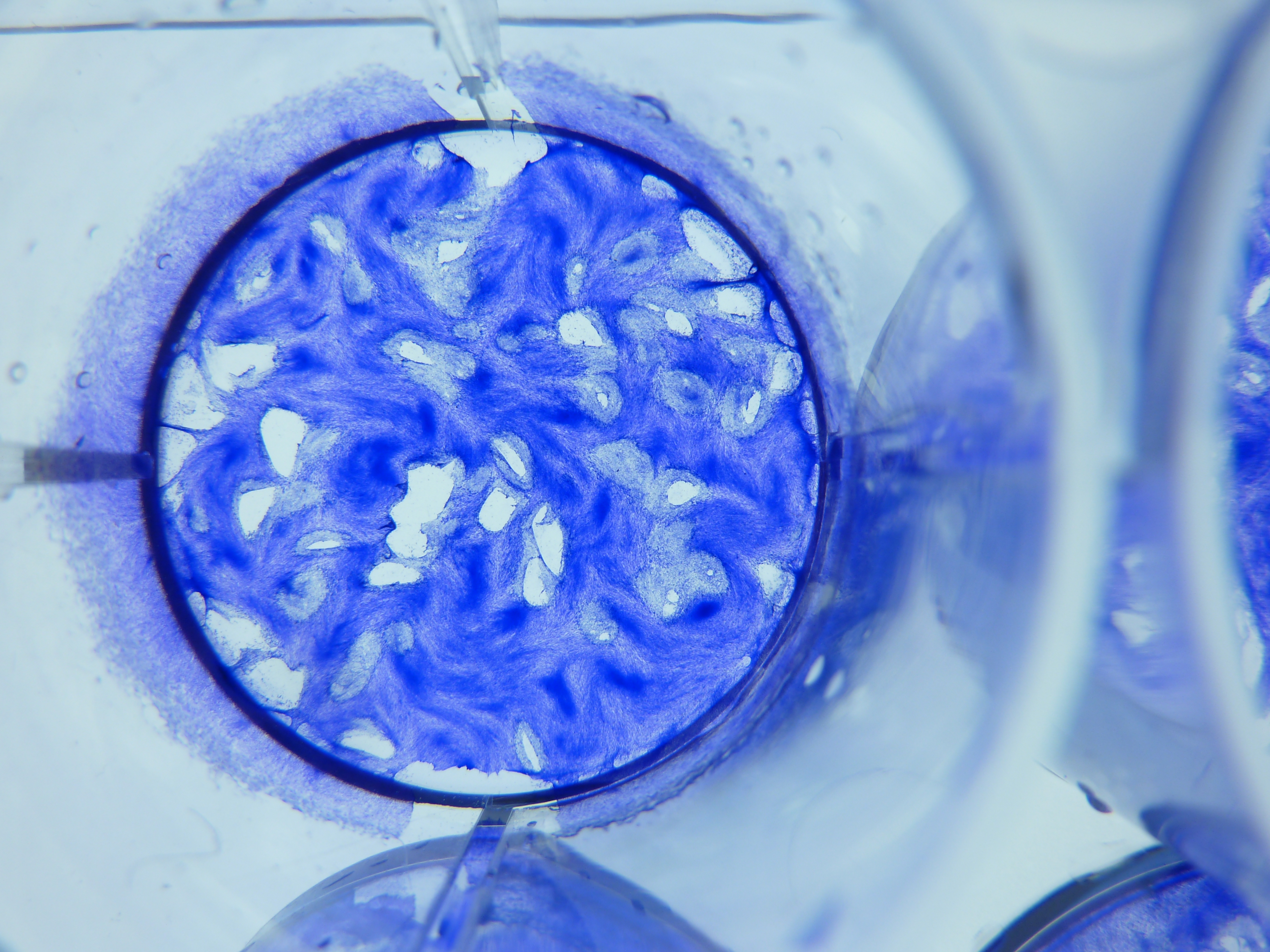

Supplement: Supplementary file 17 — Source data Fig. 9 [file 44318_2024_171_MOESM17_ESM.zip › Figure 9/9F/PA_DiCre_-Rapa.JPG]

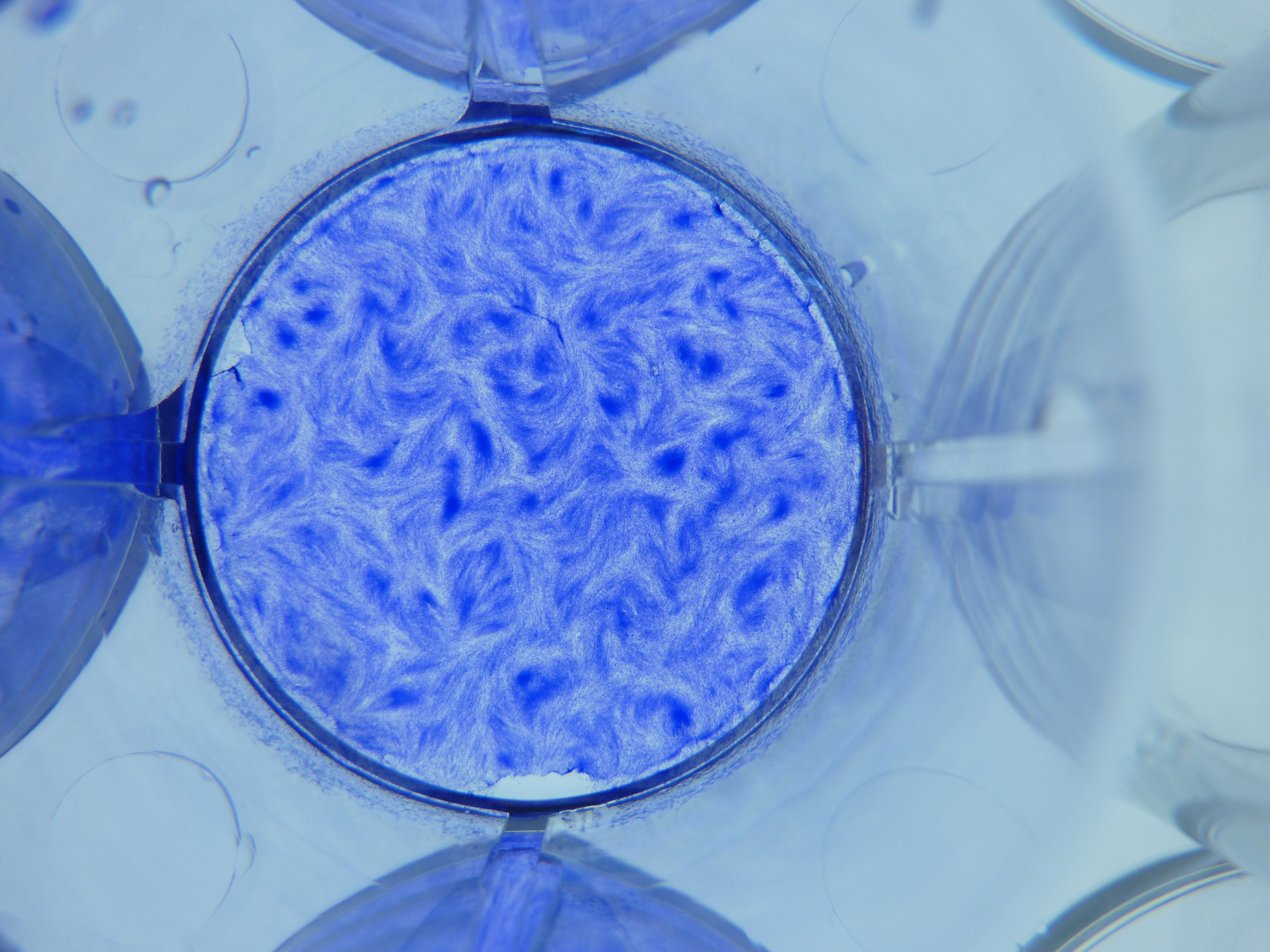

Supplement: Supplementary file 17 — Source data Fig. 9 [file 44318_2024_171_MOESM17_ESM.zip › Figure 9/9F/Pa_PP2A-C2_+Rapa.JPG]

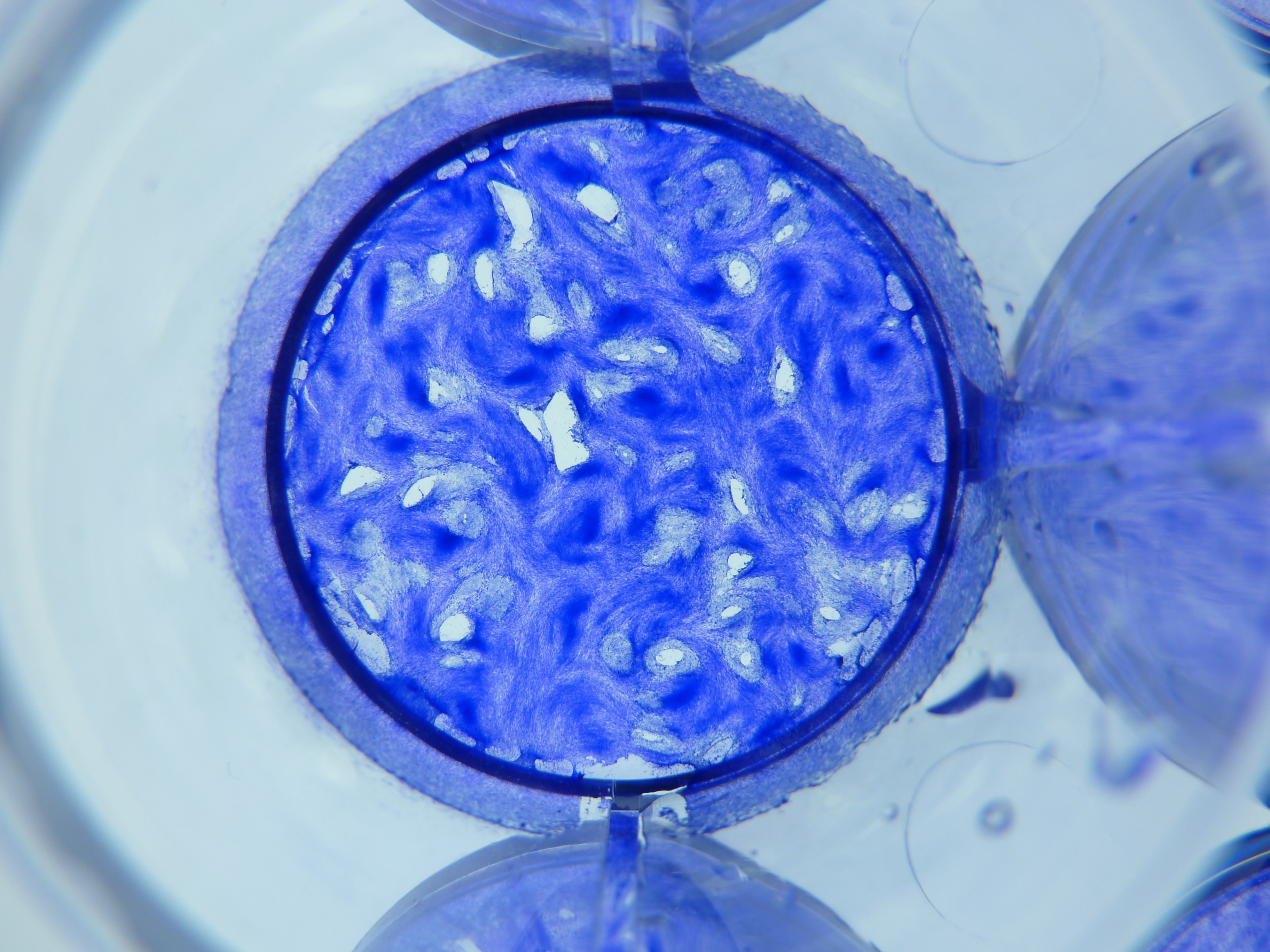

Supplement: Supplementary file 17 — Source data Fig. 9 [file 44318_2024_171_MOESM17_ESM.zip › Figure 9/9F/PA_PP2A-C2_-Rapa.JPG]
