# Supplementary material for: Cytokinetic abscission in Toxoplasma gondii is governed by protein phosphatase 2A and the daughter cell scaffold complex
Source: EMBO J. 2024 Jul 15;43(17):11. doi: 10.1038/s44318-024-00171-9 (PMC11377541; doi:10.1038/s44318-024-00171-9)
Supplement: Supplementary file 18 — Expanded View Figures [file 44318_2024_171_MOESM18_ESM.pdf]

Expanded View Figures

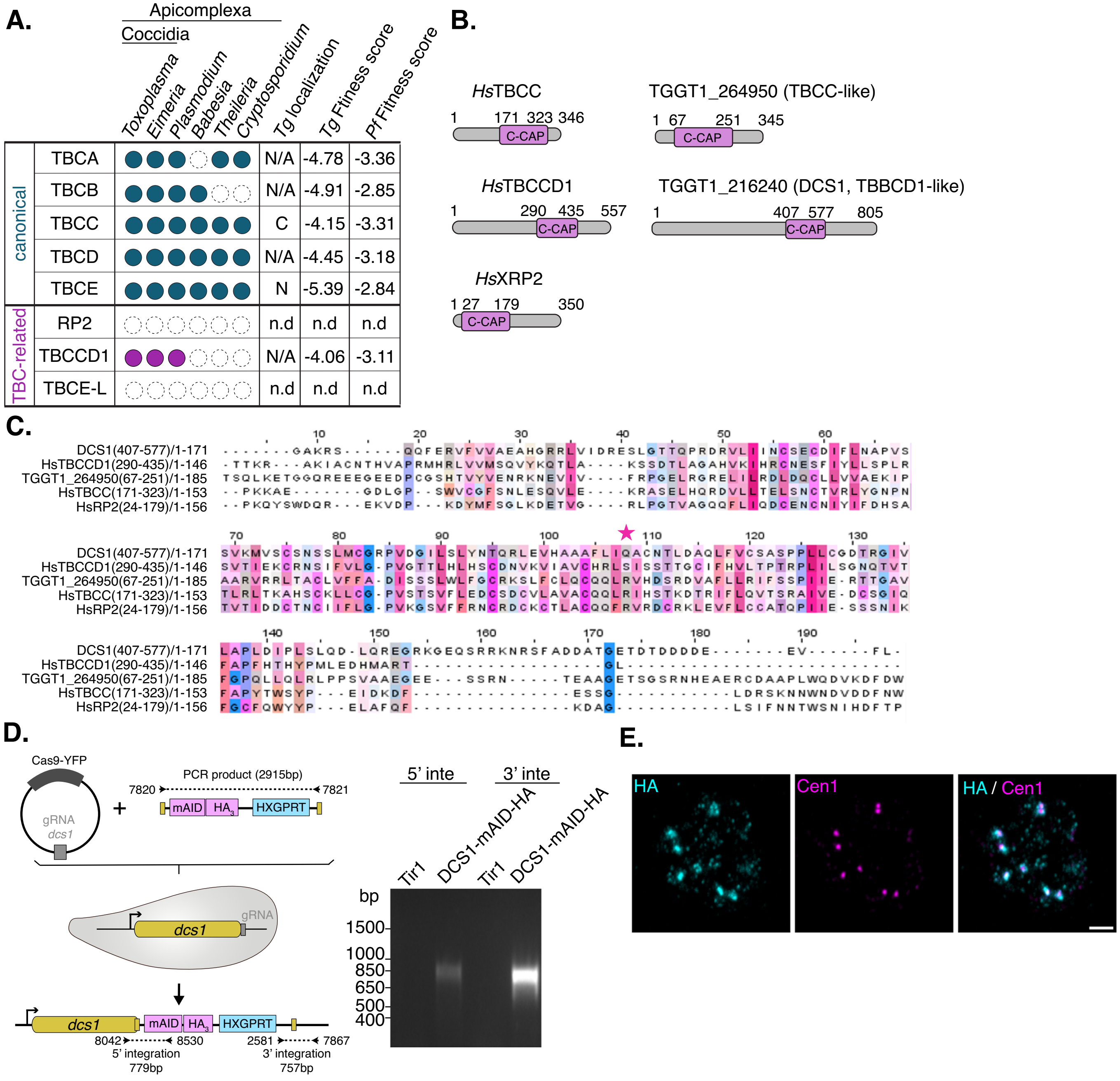

Figure EV1. DCS1 is a TBCCD1-like protein, member of the TBC family.

(**A**) Table showing the conservation of canonical TBC members and TBC-related proteins among Apicomplexa. The localization predicted by whole-cell spatial proteomics as well as the predicted gene essentiality in *T. gondii* and *P. falciparum* parasites are mentioned (Sidik et al, 2016; Zhang et al, 2018; Barylyuk et al, 2020). (**B**) Schematic representation of the domain organization of TBCC family members found in *Homo sapiens* and *T. gondii*. (**C**) Protein alignment of the C-CAP domain of TBCC family members found in *Homo sapiens* and *T. gondii*. The arginine identified as important for the GAP activity in TBCC and RP2 proteins is indicated by a pink star. The alignment was performed with Jalview 2.0 (Waterhouse et al, 2009). (**D**) Schematic of the strategy used to generate DCS1-mAID-HA transgenic parasites. The primers used to verify cassette integration are indicated and the corresponding agarose gel showing the amplification at the expected size is shown. (**E**) IFA on intracellular DCS1-mAID-HA parasites showed a partial colocalization between the duplicated centrioles (Cen1 antibodies) during DC formation. Scale bar = 2 µm.

 © The Author(s)

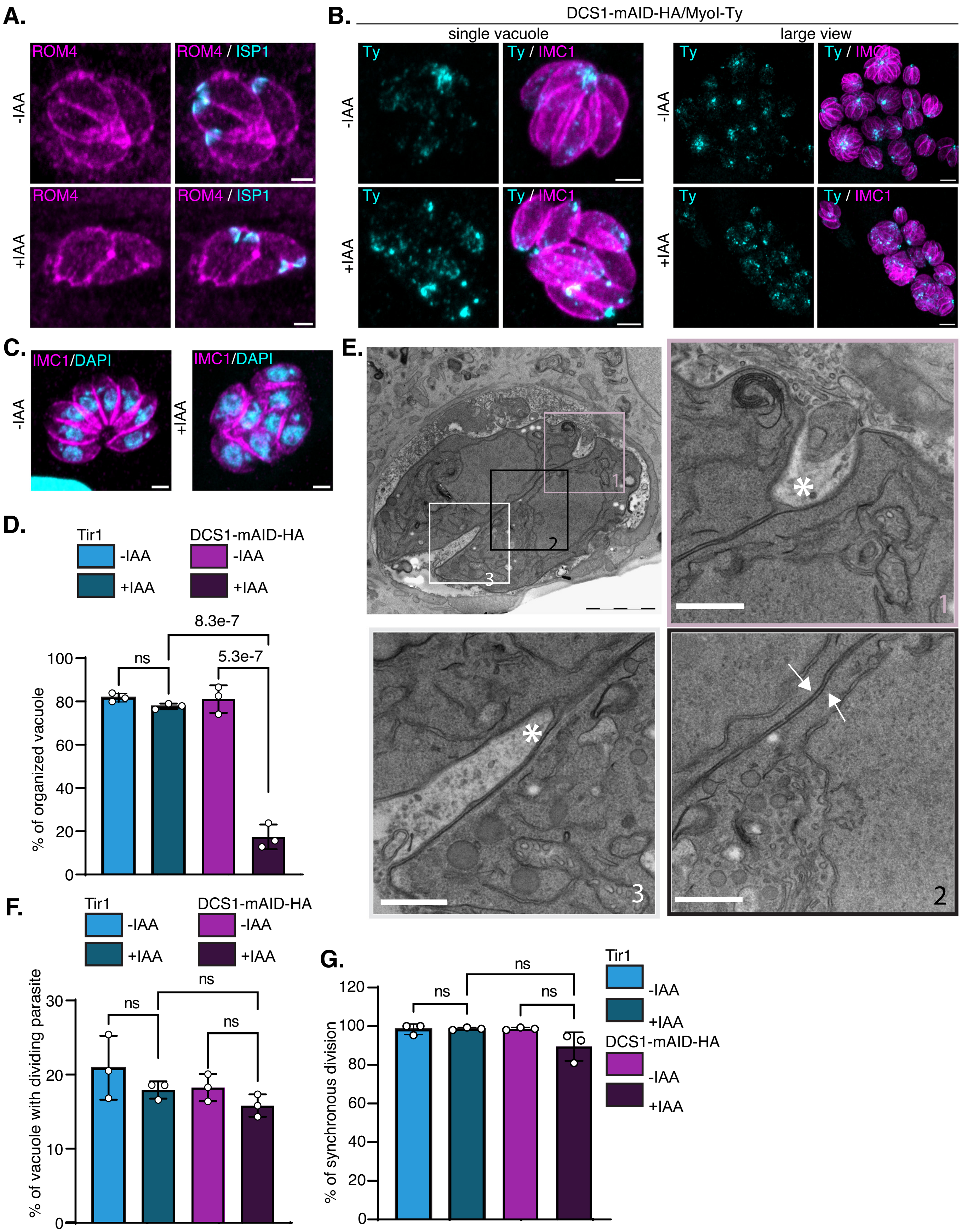
A.
ROM4
ROM4 / ISP1
-IAA
+IAA
B.
DCS1-mAID-HA/MyoI-Ty
single vacuole
large view
Ty
Ty / IMC1
C.
IMC1/DAPI
D.
Tir1
DCS1-mAID-HA
-IAA
+IAA
8.3e-7
5.3e-7
ns
% of organized vacuole
E.
F.
% of vacuole with dividing parasite
G.
% of synchronous division

Figure EV2. Incomplete cytokinesis affects parasite organization but does not compromise parasite division.

(**A**) IFA of intracellular parasites showing that cytokinesis is incomplete under DCS1 depletion using anti-ROM4 antibodies (magenta) and ISP1 antibodies (cyan). Scale bar = 2 µm. (**B**) IFA of intracellular DCS1-mAID-HA/MyoI-Ty expressing parasites and treated or not with IAA for 24 h. MyoI is detected using anti-Ty antibodies (cyan) and the parasite is visualized using anti-IMC1 antibodies (magenta). Scale bar = 2 µm in the single vacuole view and 5 µm in the large view. (**C**) IFA of intracellular parasites showing an example of organized and disorganized parasites within a vacuole. Scale bar = 2 µm. (**D**) Quantification of vacuole presenting organized parasites in different conditions. One-way ANOVA followed by Tukey's multiple comparison was used to test differences between groups (mean ± SD; $n = 3$ independent biological replicates). (**E**) Insets showing higher magnification of the electron microscopy image presented in Fig. 4D. Asterisks show the beginning of plasma membrane invagination, and the white arrows indicate the DC IMC facing each other and absence of PM. Scale bar of the insets = 1 µm. (**F**) Quantification of vacuole with parasites undergoing division using anti-IMC1 antibodies to visualize DC IMC. One-way ANOVA followed by Tukey's multiple comparison was used to test differences between groups (mean ± SD; $n = 3$ independent biological replicates). (**G**) Quantification of vacuole with parasites that are dividing synchronously. Anti-IMC1 antibodies were used to visualized DC IMC. One-way ANOVA followed by Tukey's multiple comparison was used to test differences between groups (mean ± SD; $n = 3$ independent biological replicates).

 © The Author(s)

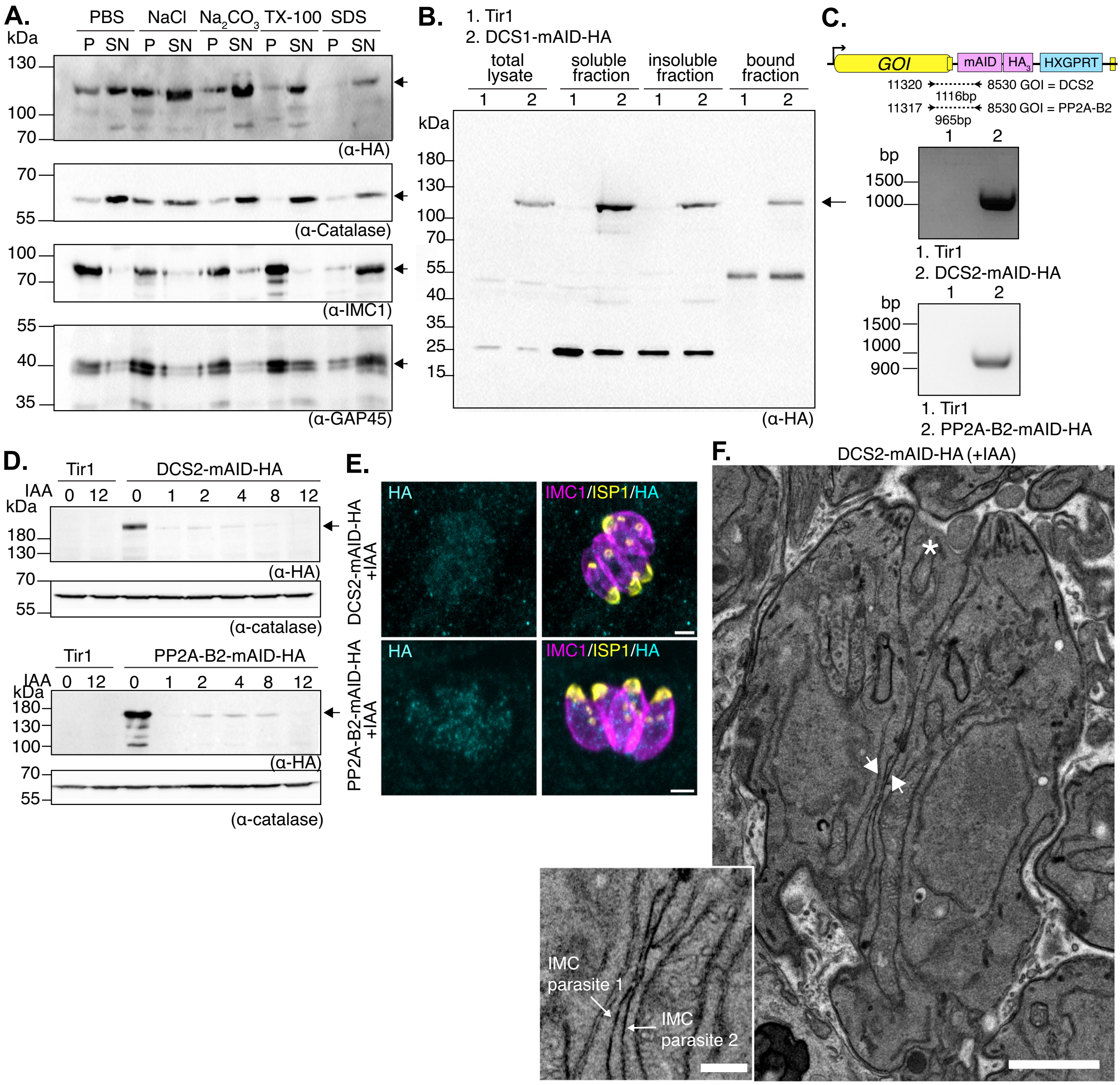

Figure EV3. Immunoprecipitation of DCS1 and generation of iKD strains for the identified DCS1-interacting partners.

(**A**) WB analysis of DCS1 solubility in different buffers (PBS, NaCl 1 M, Na_2CO_3 0.1 M, TX-100 1% and SDS 1%) accompanied with parasite lysis by freeze and thaw cycles. Catalase (anti-catalase antibodies) is a soluble cytoplasmic protein, the membrane-associated protein GAP45 (anti-GAP45 antibodies) is soluble in presence of detergent 1% TX-100 or 1% SDS. The intermediate filament-like protein IMC1 is only soluble in 1% SDS. The black arrows indicate the protein revealed by the specific antibodies used (DCS1 for anti-HA, catalase for anti-catalase, IMC for anti-IMC1 and GAP45 for anti-GAP45 antibodies). (**B**) WB analysis of the different fractions collected during the immunoprecipitation of DCS1 from DCS1-mAID-HA parasites using anti-HA antibodies. As a control, immunoprecipitation was also performed on Tir1 parasites. DCS1 (indicated by a black arrow) is mainly found in the soluble fraction and in the bound fraction only in DCS1-mAID-HA expressing parasite. (**C**) Schematic of the strategy used to generate DCS2-mAID-HA and PP2A-B2-mAID-HA transgenic parasites. The primers used to verify cassette integration are indicated and the corresponding agarose gel showing the amplification at the expected size is shown. (**D**) WB analysis of Tir1, DCS2-mAID-HA and PP2A-B2-mAID-HA parasites lysates showing efficient protein depletion following different time of IAA treatment (hours). The arrows indicate the signal corresponding to DCS2 and PP2A-B2 proteins ($n = 3$ biologically independent experiments). (**E**) IFA on intracellular DCS2-mAID-HA and PP2A-B2-mAID-HA parasites showed that HA signal (cyan) became undetectable after 24 h of IAA treatment. The apical cap and the IMC of the mother and daughter cells are detected with anti-ISP1 (yellow) and anti-IMC1 (magenta) antibodies. Scale bar = 2 µm. (**F**) Electron microscopy image of intracellular DCS2-mAID-HA treated 24 h with IAA. Asterisks show the absence of plasma membrane invagination, and the white arrows indicated the DC IMC facing each other and absence of PM. Scale bar = 1 µm. Inset shows a zoomed view of the IMC of each parasite facing each other and the absence of PM in between. Scale bar = 250 nm.

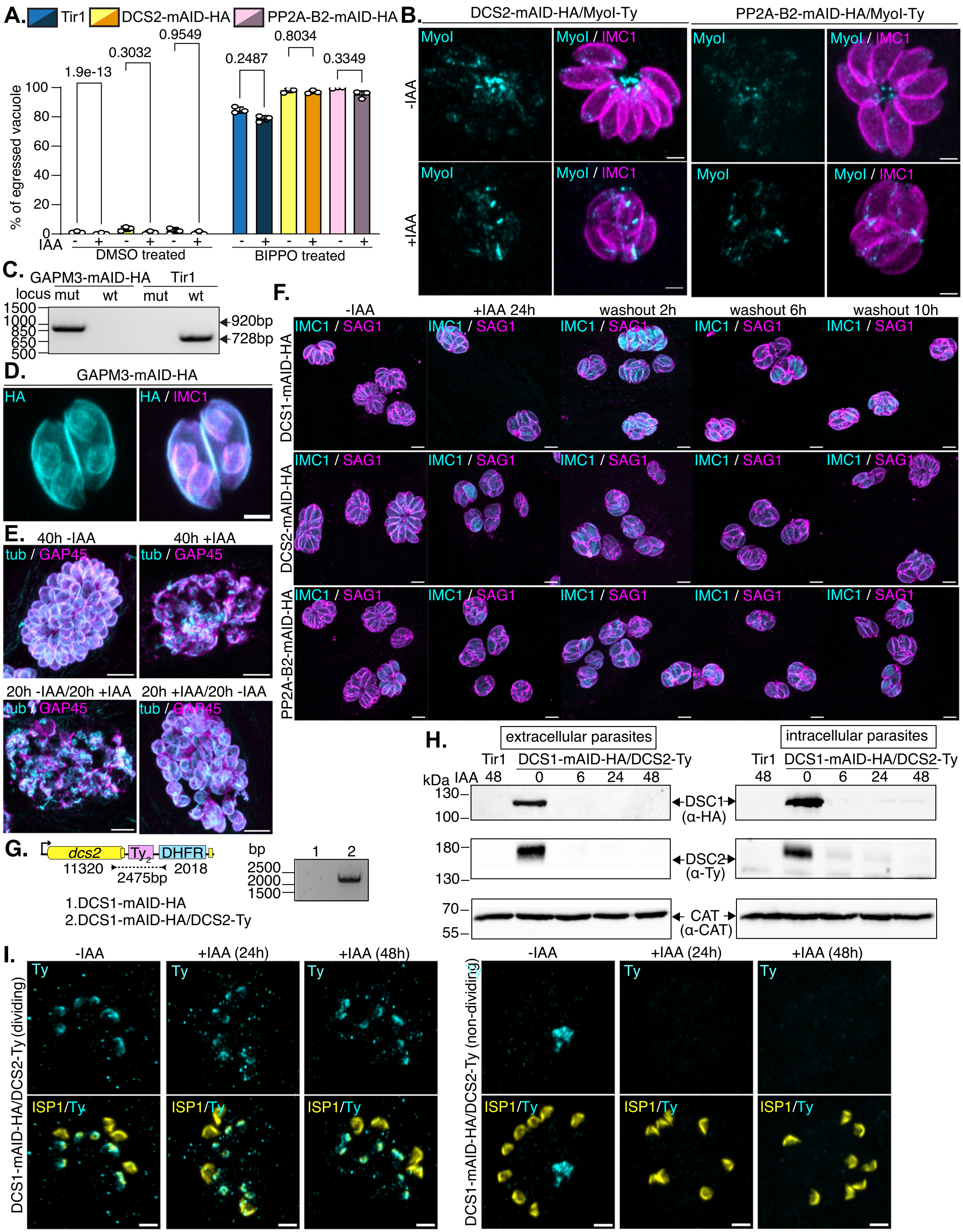
A.
Tir1
DCS2-mAID-HA
PP2A-B2-mAID-HA
% of egressed vacuole
1.9e-13
0.3032
0.9549
0.2487
0.8034
0.3349
IAA
DMSO treated
BIPPO treated
B.
DCS2-mAID-HA/MyoI-Ty
PP2A-B2-mAID-HA/MyoI-Ty
-IAA
+IAA
MyoI
MyoI / IMC1
C.
GAPM3-mAID-HA
Tir1
locus mut wt mut wt
920bp
728bp
D.
GAPM3-mAID-HA
HA
HA / IMC1
E.
40h -IAA
40h +IAA
20h -IAA/20h +IAA
20h +IAA/20h -IAA
tub / GAP45
F.
-IAA
+IAA 24h
washout 2h
washout 6h
washout 10h
IMC1 / SAG1
DCS1-mAID-HA
DCS2-mAID-HA
PP2A-B2-mAID-HA
G.
dcs2
Ty2
DHFR
11320
2475bp
2018
bp
1.DCS1-mAID-HA
2.DCS1-mAID-HA/DCS2-Ty
H.
extracellular parasites
intracellular parasites
Tir1
DCS1-mAID-HA/DCS2-Ty
kDa IAA 48 0 6 24 48
DSC1
(α-HA)
DSC2
(α-Ty)
CAT
(α-CAT)
I.
-IAA
+IAA (24h)
+IAA (48h)
Ty
ISP1/Ty
DCS1-mAID-HA/DCS2-Ty (dividing)
DCS1-mAID-HA/DCS2-Ty (non-dividing)

 © The Author(s)

Figure EV4. Immunoprecipitation of DCS1 and generation of new strains regarding the identified DCS1-interacting partners.

(**A**) Graph representing the percentage of ruptured vacuoles following treatment with the egress inducer BIPPO for DCS2- and PP2A-B2-mAID-HA strains. Two-way ANOVA followed by Tukey's multiple comparison was used to test differences between groups (mean ± SD; $n = 3$ biologically independent experiments). (**B**) IFA of intracellular DCS2-mAID-HA/MyoI-Ty or PP2A-B2-mAID-HA/MyoI-Ty expressing parasites and treated or not with IAA for 24 h. MyoI is detected using anti-Ty antibodies (cyan) and the parasite is visualized using anti-IMC1 antibodies (magenta). Scale bar = 2 µm. (**C**) Integration PCR of GAPM3-mAID-HA parasites. Primers 12017/12018 were used to amplify the wt locus while the primer pair 12017/7139 were used to assess proper integration of the mAID-HA cassette at the endogenous locus. Parental Tir1 strain was used as a control. (**D**) IFA on intracellular parasites showing that GAPM3 (cyan) is localized at the IMC (IMC1 antibodies—magenta) of the mother as well as of the DC parasites as previously described (Harding et al, 2019). Scale bar = 2 µm. (**E**) The destabilization of subpellicular microtubules induced by GAPM3 depletion is reversible as shown by IFA. Subpellicular MTs are detected with anti-tubulin antibodies (cyan) and the IMC with GAP45 antibodies (magenta). Scale bar = 5 µm. (**F**) IFA showing the reversibility of the cellular abscission defect observed under DCS1, DCS2, or PP2A-B2 depletion (induced by 24 h of IAA treatment) at different time post-washout of IAA. Parasite IMC1 is shown using IMC1 antibodies (cyan) and the plasma membrane is visualized using SAG1 antibodies (magenta). Scale bar = 5 µm. (**G**) Schematic of the strategy used to generate DCS1-mAID-HA/DCS2-Ty transgenic parasites. The primers used to verify cassette integration are indicated and the corresponding agarose gel showing the amplification at the expected size is shown. (**H**) WB analysis of intracellular and extracellular DCS1-mAID-HA/DCS2-Ty parasites lysates treated or not with IAA for the indicated time. DCS1 and DCS2 protein levels were detected using anti-HA and anti-Ty antibodies. Catalase was used as a loading control ($n = 3$ biologically independent experiments). (**I**) IFA analysis of intracellular DCS1-mAID-HA/DCS2-Ty parasites treated or not with IAA for 24 h and 48 h to deplete DCS1. Dividing parasites (left panel) and non-dividing parasites (right panel) are depicted. DCS2 is visualized using anti-Ty antibodies (cyan) and the apical cap of the parasite is stained with anti-ISP1 antibodies (yellow). Scale bar = 2 µm.

© The Author(s)

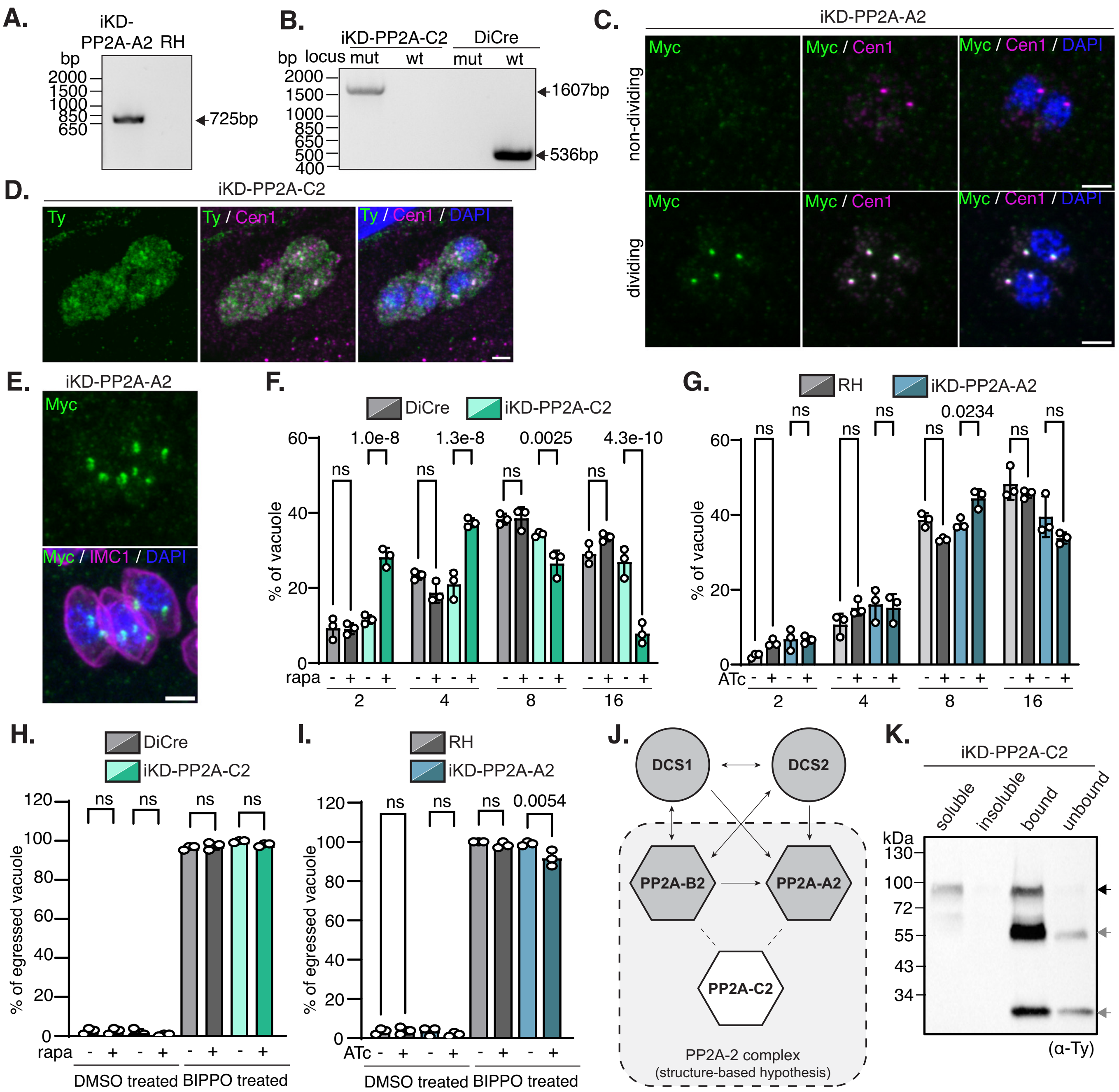
A.
iKD-PP2A-A2
RH
bp
2000
1500
1000
850
650
725bp
B.
iKD-PP2A-C2
DiCre
bp locus mut wt mut wt
1607bp
536bp
C.
iKD-PP2A-A2
non-dividing
dividing
Myc
Myc / Cen1
Myc / Cen1 / DAPI
D.
iKD-PP2A-C2
Ty
Ty / Cen1
Ty / Cen1 / DAPI
E.
iKD-PP2A-A2
Myc
Myc / IMC1 / DAPI
F.
DiCre
iKD-PP2A-C2
% of vacuole
rapa
1.0e-8
1.3e-8
0.0025
4.3e-10
G.
RH
iKD-PP2A-A2
0.0234
ATc
H.
% of egressed vacuole
DMSO treated BIPPO treated
I.
0.0054
J.
DCS1
DCS2
PP2A-B2
PP2A-A2
PP2A-C2
PP2A-2 complex
(structure-based hypothesis)
K.
soluble
insoluble
bound
unbound
kDa
(α-Ty)

 © The Author(s)

Figure EV5. Subunits of PP2A-2 complex localized to the centrosome and support late cytokinesis.

(**A**) Integration PCR showing the integration of the tetR cassette at the PP2A-A2 endogenous locus. Primer pair 12019/3596 was used to amplify the recombinant locus. The parental RHΔKu80 strain was used as a control. (**B**) Integration PCR showing the integration of the U1 cassette at the PP2A-C2 endogenous locus. Primer pair 11999/12000 was used to amplify the recombinant locus (mut) while the primer pair 11999/4610 was used to amplify the wt locus. The parental DiCre strain was used as a control. (**C**) IFA on iKD-PP2A-A2 parasites showing the colocalization of the protein (Myc antibodies—green) with the centrosome (Cen1 antibodies—magenta) in dividing and non-dividing parasites. Nucleus is visualized with DAPI. Scale bar = 2 µm. (**D**) IFA on intracellular iKD-PP2A-C2 parasites showing the colocalization of the protein (Ty antibodies—green) with the centrosome (Cen1 antibodies—magenta). Nucleus is visualized with DAPI. Scale bar = 2 µm. (**E**) IFA on iKD-PP2A-A2 parasites showing the appearance of PP2A-A2 (Myc antibodies—green) prior DC bud assembly (IMC1 antibodies—magenta). Nucleus is visualized with DAPI. Scale bar = 2 µm. (**F**) Graph representing the number of parasites per vacuole observed at 30 h post-invasion for parental DiCre or iKD-PP2A-C2 strains treated or not with rapamycin. Two-way ANOVA followed by Tukey's multiple comparison was used to test differences between strains for each category (mean ± SD; $n = 3$ biologically independent experiments). (**G**) Graph representing the number of parasites per vacuole observed at 30 h post-invasion for parental RHΔKu80 or iKD-PP2A-A2 strains treated or not with ATc. Two-way ANOVA followed by Tukey's multiple comparison was used to test differences between strains for each category (mean ± SD; $n = 3$ biologically independent experiments). (**H**) Graph representing the percentage of ruptured vacuoles following treatment with the egress inducer BIPPO for DiCre and iKD-PP2A-C2 strains treated for 30 h with rapamycin. Two-way ANOVA followed by Tukey's multiple comparison was used to test differences between groups (mean ± SD; $n = 3$ biologically independent experiments). (**I**) Graph representing the percentage of ruptured vacuoles following treatment with the egress inducer BIPPO for RHΔKu80 or iKD-PP2A-A2 strains treated for 30 h with ATc. Two-way ANOVA followed by Tukey's multiple comparison was used to test differences between groups (mean ± SD; $n = 3$ biologically independent experiments). (**J**) Interactome of DCS and PP2A proteins identified based on co-immunoprecipitation and structural homology results. Arrows points toward co-immunoprecipitated partners. (**K**) WB analysis of the immunoprecipitation performed on iKD-PP2A-C2 parasites using anti-Ty antibodies. PP2A-C2 is mainly found in the bound fraction (black arrow). Heavy- and light chain of the antibodies used are indicated by the grey arrows.

© The Author(s)
